# Autophagy mediates grain yield and nitrogen stress resistance by modulating nitrogen remobilization in rice

**DOI:** 10.1371/journal.pone.0244996

**Published:** 2021-01-14

**Authors:** Xiaoxi Zhen, Naimeng Zheng, Jinlei Yu, Congyuan Bi, Fan Xu

**Affiliations:** 1 Key Laboratory of Northern Japonica Rice Genetics and Breeding, Ministry of Education and Liaoning Province, Key Laboratory of Northeast Rice Biology and Genetics and Breeding, Ministry of Agriculture, Rice Research Institute of Shenyang Agricultural University, Shenyang, China; 2 College of Agriculture, Shanxi Agricultural University, Taigu, China; Hainan University, CHINA

## Abstract

Autophagy, a conserved cellular process in eukaryotes, has evolved to a sophisticated process to dispose of intracellular constituents and plays important roles in plant development, metabolism, and efficient nutrients remobilization under suboptimal nutrients conditions. Here, we show that *OsATG8b*, an *AUTOPHAGY-RELATED8* (*ATG8*) gene in rice, was highly induced by nitrogen (N) starvation. Elevated expression of *OsATG8b* significantly increased ATG8 lipidation, autophagic flux, and grain yield in rice under both sufficient and deficient N conditions. Overexpressing of *OsATG8b* could greatly increase the activities of enzymes related to N metabolism. Intriguingly, the ^15^N-labeling assay further revealed that more N was remobilized to seeds in *OsATG8b*-overexpressing rice, which significantly increased the N remobilization efficiency (NRE), N harvest index, N utilization efficiency (NUE), and N uptake efficiency (NUpE). Conversely, the *osatg8b* knock-out mutants had the opposite results on these characters. The substantial transcriptional changes of the overexpressed transgenic lines indicated the presence of complex signaling to developmental, metabolic process, and hormone, etc. Excitingly, the transgenic rice under different backgrounds all similarly be boosted in yield and NUE with *OsATG8b* overexpression. This work provides an excellent candidate gene for improving N remobilization, utilization, and yield in crops simultaneously.

## Introduction

Autophagy (self-eating) is an ancient and universal process for degrading and removing cellular debris, which consists of five stages: initiation, the formation of autophagosomes, expansion, engulfment of cargoes, and delivery of the cargo for degradation [1]. It plays an essential role in the maintenance of cellular homeostasis [2]. The *AUTOPHAGY-RELATED* (*ATG*) gene was originally identified by Yoshinori Ohsumi in yeast [3]. Subsequently, a large number of plant autophagy genes are characterized from the orthologs of yeast *ATG*, such as Arabidopsis (*Arabidopsis thaliana*), rice (*Oryza sativa*), maize (*Zea mays*), tobacco (*Nicotiana tabacum*), foxtail millet (*Setaria italica*) and the alga (*Chlamydomonas reinhardtii*) [4–9]. It was shown that a variety mutants of Arabidopsis, maize, and rice displayed growth inhibition, accelerated leaf senescence, hypersensitivity to nutrition starvation, and reduced fecundity and yield [10–15]. Typically, these phenotypes are more pronounced when mutants are grown under nutrient-deficient conditions.

N is one of the main determinants for crop yield formation, breeding cultivars with high NUE has been an urgent requirement for sustainable crop productivity [16]. However, plant NUE is complex and regulated by multiple interacting genetic and environmental factors [17, 18]. Autophagy is essential for N remobilization and reuse, which affects the yield and quality of grain in cereal crops, especially under N deficiency stress [19]. A study of the Arabidopsis *atg5*, *atg9*, and *atg18a* mutants first evidenced that the autophagy regulated N remobilization from leaves to seeds [20]. It was subsequently found that the NUE in *atg5* mutant seeds sharply decreased to about 50% of that in the wild-type seeds under N restriction [20]. Similar results were found in studies of crops such as rice and maize, the biomass and NUE of rice *osatg7-1* mutant were strongly affected during the vegetative growth stage [14]. ^15^N partitioning studies revealed that the amount of recycled N exported in maize *atg12* mutant was substantially decreased by two-fold [5]. Using a multi-omics strategy, McLoughlin showed that autophagy also significantly affects the remodeling of the maize proteome and lipids [21]. A relationship between autophagy and the deficiency of other nutrients (P, S, and Zn) has also been found [22–24], but the role of autophagy under these conditions has not been clearly defined.

Among the many plant ATG proteins, ATG8 is a key protein and central player involved in the trafficking and vacuolar fusion of autophagosomes because it provides a docking site for ATG8 interaction motif (AIM)-containing autophagic receptors that selectively recruit cargo [25]. The interaction of ATG8 and autophagy receptors leads to the formation of autophagosomes [26, 27]. However, there is also evidence that ATG8-independent autophagy occurs in maize aleurone cells, delivering storage proteins to novel vacuoles [28]. Jia et al. identified an unrecognized conserved senescence regulatory pathway controlled by ATG8-ABS3-mediated proteostasis, which is not dependent on autophagy in wheat [29].

There is a single ATG8 in yeast and other fungi, but there are several ATG8 isoforms with redundant functions in higher eukaryotes [30–32]. Intriguingly, several studies found that overexpression of *ATG8* homologs in transgenic plants not only led to better resistance to stresses and starvations, but also accelerated growth under optimal growth conditions, such as overexpression of *AtAtg8f* in Arabidopsis could promote the growth, increased rosettes size and conferred tolerance to N and C limitation [33]. Overexpression of apple *MdATG8i* in Arabidopsis could promote plant growth, advanced bolting, accelerated leaf senescence, and enhanced tolerance to N and C starvation, while in apple callus cells could accelerate growth and enhanced N starvation tolerance [34]. Overexpression of soybean *GmATG8c* in Arabidopsis could promote plant growth, advanced bolting, increased yield and tolerance to N and C starvation, while in soybean callus could enhance N starvation tolerance and accelerate the growth of the calli [35]. Foxtail millet *SiATG8a* overexpressing in Arabidopsis could increase resistance to both drought stress and N starvation [36], while in rice could confer tolerance to N starvation [6]. The recent study also showed that overexpression of *AtATG8a*, *AtATG8e*, *AtATG8f*, or *AtATG8g* in Arabidopsis could improve seed N% and reduce N waste in dry remains under full nitrate conditions, though there was little difference in plant phenotype [37]. Seven ATG8 isoforms (*OsATGa* to *OsATGg*) have been identified in the rice genome. The first cloned rice autophagy associated gene was *OsATG8a*, which interacted with *OsATG4* [38]. *OsATG8a* to *OsATG8c* share high levels of amino acid sequence identity, and *OsATG8d* is highly homologous to *AtATG8i* [8, 9]. The mRFP-OsATG8a and mRFP-OsATG8d fusions were used as autophagy markers to monitor autophagy directly [39]. *OsATG8e* has a gene locus but lacks supporting expressed sequence tag (EST) data, and *OsATG8f* has no corresponding full-length cDNA, while *OsATG8i* has not been mapped to the rice genome [8]. Although these research reported the *OsATG8*s of rice, whose physiological and molecular functions are still not clear in rice, except for our own research showed that overexpression of *OsATG8a* or *OsATG8c* caused higher NUE and yield in rice [40, 41].

In this study, we found that constitutive overexpression of *OsATG8b* in rice enhanced the autophagic flux and grain yield, leading to the strong stimulation of growth and N stress resistance. N partitioning studies showed that overexpression of *OsATG8b* significantly enhanced N harvest index (NHI) and NUE, and in a ^15^N isotope tracer experiment the ^15^N allocation to seeds was significantly higher than that in the control group. In contrast, *osatg8b* mutants showed opposite effects to those of transgenic overexpression lines. The global gene expression analysis suggested key transcriptional changes associated with *OsATG8b*-mediated up-regulated autophagy. Our findings reveal that accelerating nutrient mobilization via *OsATG8b*-mediated autophagy could have agronomic benefits and suggest that *OsATG8b* is an important candidate gene for synergistically enhancing grain yield and NUE in rice.

## Results

### N-depletion induced *OsATG8b* expression and autophagosomes formation

**Fig 1:**
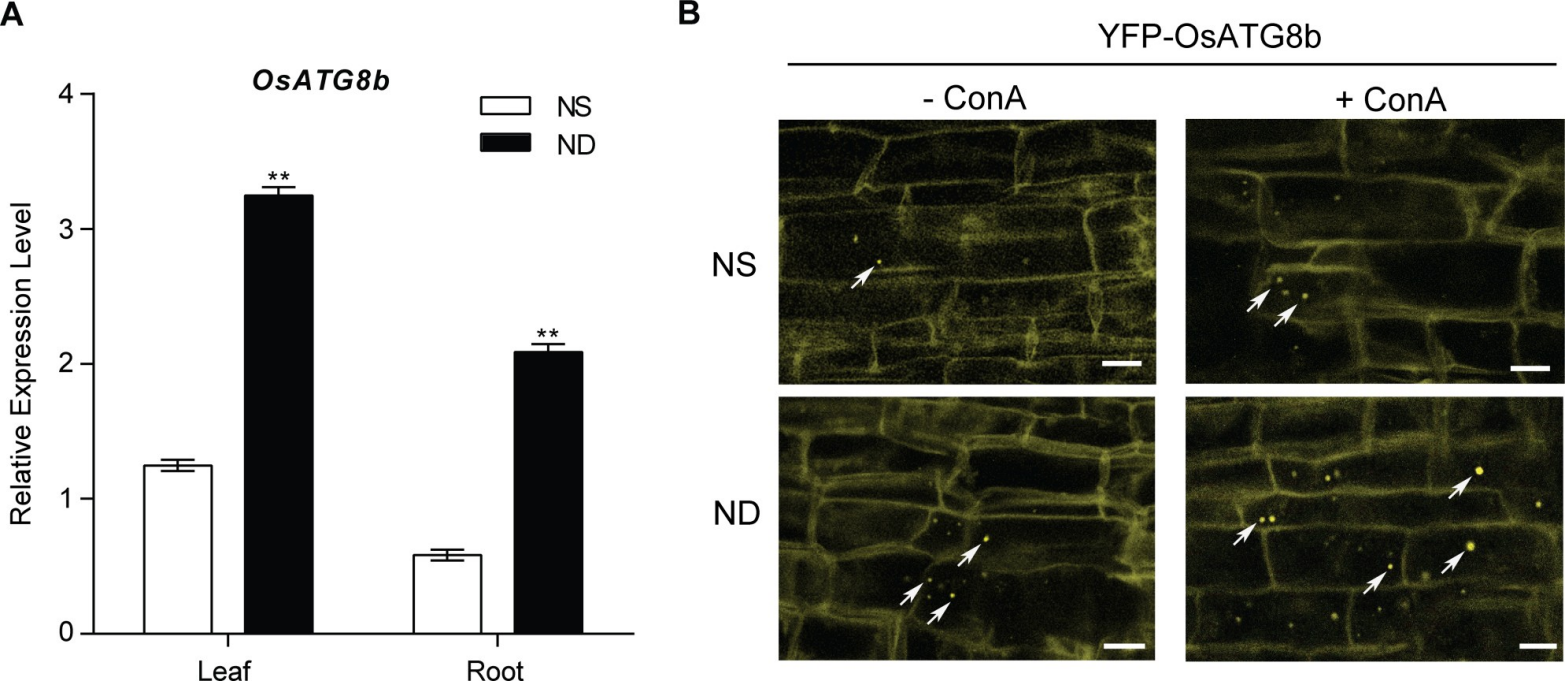
Nitrogen-induced expression pattern of *OsATG8b* gene and confocal analysis of YFP-OsATG8b in rice. (A) 14-day-old rice seedlings of SN9816 cultured with NS solution transferred to the same NS (2.88 mM N) solution and the ND (0 mM N) solution for 24h (n = 10), the expression of *OsATG8b* gene in leaf and root. *OsActin1* was used as an internal control. Values are means ± SD (n = 3), **P < 0.01 (*t*-test). (B) Seedlings were grown under NS (2.88 mM N) or ND (0 mM N) conditions with or without 0.5 μM ConA for 14h. Representative confocal microscopy images of the epidermal root cells expressing YFP-OsATG8b in SN9816. The white arrowheads indicate the YFP-OsATG8b labeled autophagosomes. Scale bars, 10 μm.

To confirm whether *OsATG8b* is expressed in response to N starvation, Shennong9816 (SN9816) rice seedlings were placed under no N (ND) condition for 24 h, the expression levels of *OsATG8b* in leaves and roots were significantly induced (Fig 1A). We produced transgenic rice expressing the YFP-OsATG8b fusion protein to visualize the formation of autophagosomes in response to N-depletion. Concanamycin A (ConA) is a vacuolar acidification inhibitor that blocks the last stages of autophagy by blocking the acidification of lysosomes to facilitate observation of autophagosomes [42, 43]. Compared with N sufficient (NS, 2.88 mM N) condition, we found a significantly increased number of YFP-OsATG8b labeled autophagosomal structures in root cells with or without ConA under the ND treatment (Fig 1B). The fluorescence ratio (positive signal/area) of YFP-OsATG8b labeled autophagosomal structures in roots under ND condition was 3.25 times more than that under NS condition, while 2.42 or 5.49 times more under NS or ND conditions with application of ConA than that under NS condition. These results indicated that N-depletion induced *OsATG8b* expression and autophagosomes formation.

### Overexpression of *OsATG8b* increased autophagic flux in rice

**Fig 2:**
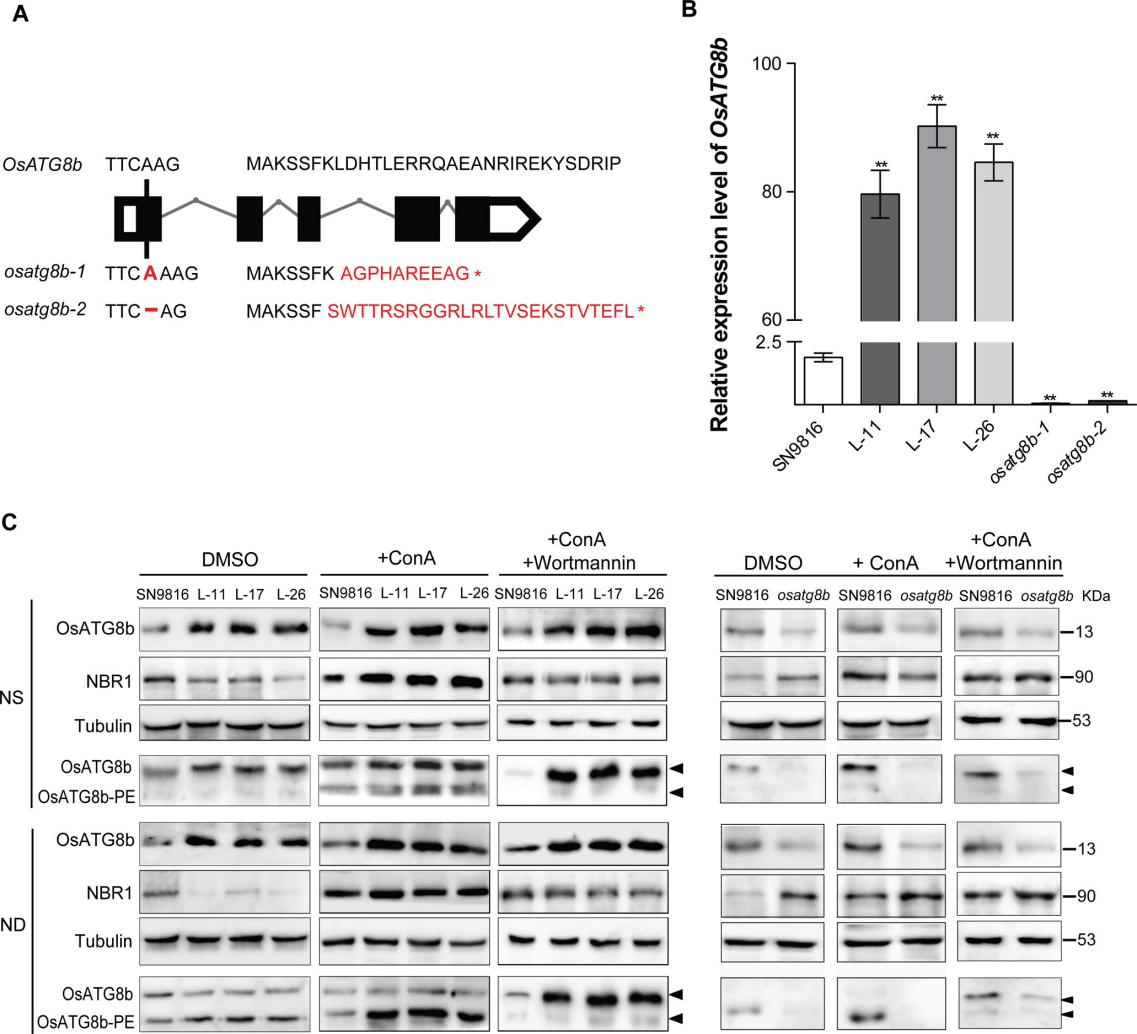
Altered expression of *OsATG8b* regulated autophagy levels in rice. (A) Targets of gene knock-out for *OsATG8b* in SN9816 with CRISPR/Cas9 system and identification of the *osatg8b* mutants by sequencing of the target sites. (B) The expression level of *OsATG8b* in leaves of SN9816, *OsATG8b*-overexpressing lines, and *osatg8b* mutant. *OsActin1* was used as an internal control. Values are means ± SD (n = 3), **P < 0.01 (*t*-test). (C) 14-day-old rice seedlings of SN9816, *OsATG8b*-overexpressing lines, and *osatg8b* mutants cultured with NS or ND solution with DMSO or 0.5 μM ConA with or without 5 μM wortmannin for 24h (n = 10), immunoblot analysis the accumulation of OsATG8b and NBR1 with anti-OsATG8b and anti-AtNBR1, near-equal protein loads were confirmed by immunoblot analysis with an α-tubulin antibody. The membrane fraction was used to detect the level of lipidated (OsATG8b-PE) and free OsATG8b in the leaves.

To investigate the function of *OsATG8b* in rice, we obtained more than 10 *OsATG8b*-overexpressing transgenic lines in SN9816 background, lines 11, 17, and 26 (L-11, L-17, and L-26) were selected as representative overexpressed lines. Additionally, we knocked out the *OsATG8b* sequence in SN9816 using the CRISPR/Cas9 system and obtained two independent mutants with different targets. Sequencing of genomic DNA in mutants *osatg8b-1* or *osatg8b-2* showed that there was a single base insertion or deletion in the first exon (18th bp) of *OsATG8b* to make a termination codon (Fig 2A). The expression of *OsATG8b* was up-regulated in *OsATG8b*-overexpressing lines, but dramatically down-regulated in *osatg8b* mutants compared with the control plant SN9816 (Fig 2B). To eliminate the influence of altered *OsATG8b* expression on other *OsATG8* genes, we also detected the expression of other *OsATG8*s. It was shown that the transcript levels of the other *OsATG8*s were not affected in *OsATG8b*-overexpressing lines, but down-regulated in *osatg8b* mutants (S1 Fig). The OsATG8b protein level was significantly increased in *OsATG8b*-overexpressing lines, while reduced in *osatg8b* mutants than in SN9816 under both NS and ND conditions using anti-OsATG8b (Fig 2C).

Since the Arabidopsis autophagy receptor Neighbor of BRCA1 (NBR1) is an autophagy substrate degraded in the vacuole, the amount of NBR1 protein degradation can be used as a measure of selective autophagic flux in plants [42, 44]. As shown in Fig 2C, NBR1 protein accumulation was lower in *OsATG8b*-overexpressing lines, but increased in *osatg8b*, especially under the ND treatment. Wortmannin is an early autophagic inhibitor that dampens autophagosome formation in plants [45]. However, the accumulation of NBR1 protein was greatly increased in *OsATG8b*-overexpressing lines when treatment with ConA, (Fig 2C, +ConA), and this process could be suppressed with the addition of wortmannin (Fig 2C, +ConA+Wortmannin), but there was no significant effect on the protein levels in *osatg8b*. It was shown that the accumulation of OsATG8b-PE was significantly increased in of *OsATG8b-*overexpressing lines, thus the OsATG8b lipidation was enhanced, especially under ND condition (Fig 2C, DMSO), and increased to a greater extent after ConA treatment (Fig 2C, +ConA), but suppressed by the wortmannin (Fig 2C, +ConA+Wortmannin). However, the accumulation of OsATG8b-PE was scarcely in the *osatg8b* mutants both under two N conditions with or without ConA or wortmannin. Although the anti-OsATG8b antibody could not distinguish the OsATG8a, OsATG8b, and OsATG8c (S2 Fig), the transcriptional expression levels of *OsATG8a* and *OsATG8c* were not affected in *OsATG8b*-overexpressing lines, and the overall autophagy flux was greatly enhanced, so we speculated that these results still related to the overexpression of *OsATG8b*. These results revealed that overexpression of *OsATG8b* significantly increases autophagic flux in transgenic rice, especially under N starvation.

### Enhanced autophagy promoted photosynthesis and N metabolism in rice

**Fig 3:**
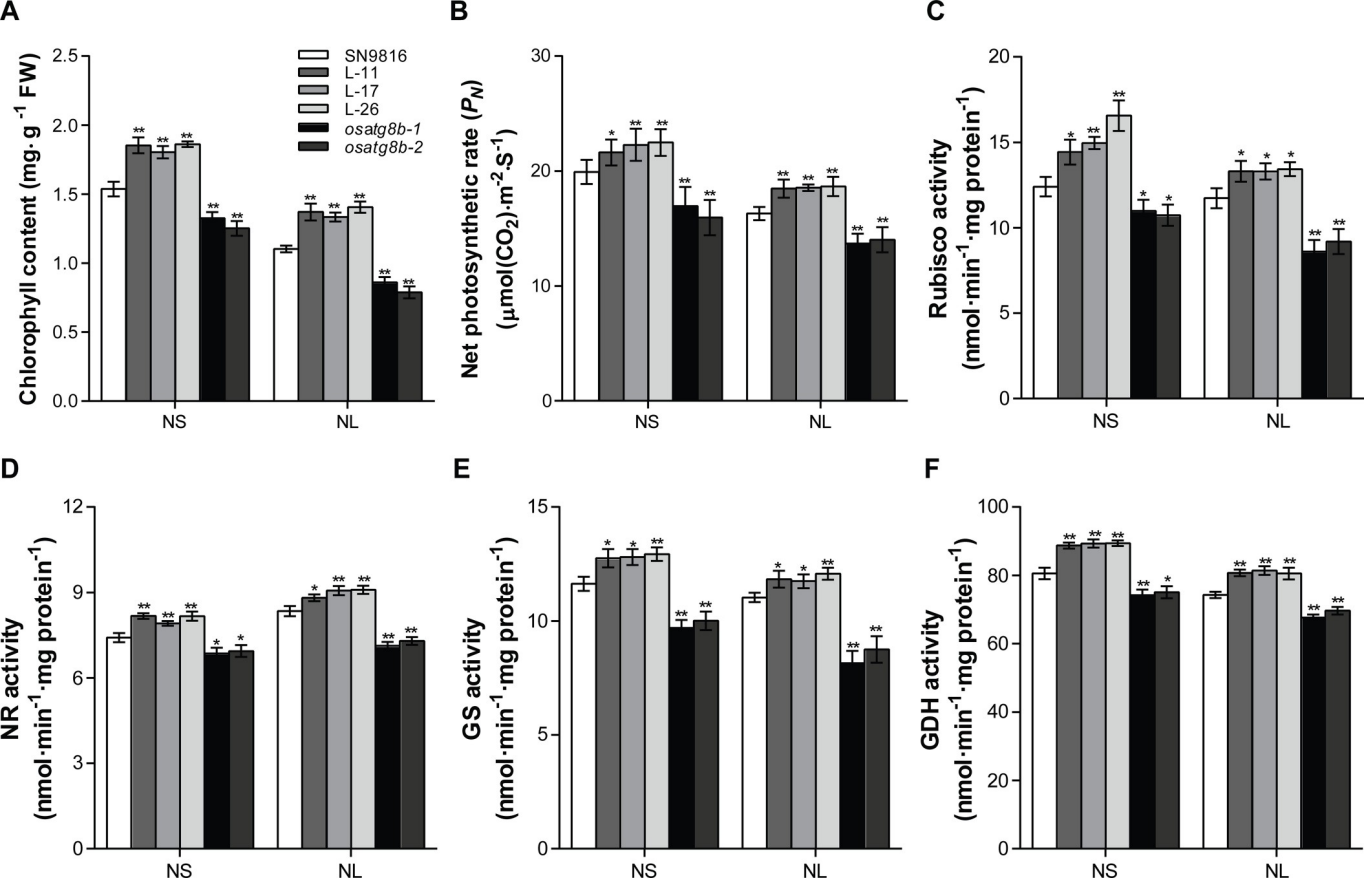
*OsATG8b* gene affected the chlorophyll content, photosynthetic character, and enzyme activity involved in photosynthesis and N metabolism. (A) The chlorophyll content, (B) Net photosynthetic rate (*P*_N_), (C) Rubisco activity, (D) Nitrate reductase (NR) activity, (E) Glutamine synthetase (GS) activity, and (F) Glutamate dehydrogenase synthesis (GDH) in flag leaves of SN9816, *OsATG8b*-overexpressing lines and *osatg8b* mutants under NS (225 kg·ha^-1^) and NL (75 kg·ha^-1^) conditions at the grain-filling stage. Values are means ± SD (n = 12), *P < 0.05, **P < 0.01 (*t*-test).

We further investigated the physiological function of *OsATG8b* in the flag leaves at the grain-filling stage. It was shown that the chlorophyll content, net photosynthetic rate (*P*_N_) and Rubisco activity in *OsATG8b*-overexpressing lines were significantly higher than that of SN9816 under NS (225 kg·ha^-1^) (increased by an average of 20.13%, 8.51%, and 23.47%, respectively) and NL (75 kg·ha^-1^) (increased by an average of 24.5%, 13.87%, and 13.82%, respectively) conditions, but dramatically decreased in *osatg8b* mutants under both two N conditions (Fig 3A–3C). Given that *OsATG8b*-overexpressing lines performed better under both NS and NL conditions, we speculated that *OsATG8b* may affect N metabolism. Compared with SN9816, the activities of nitrate reductase (NR), glutamine synthetase (GS) and glutamate dehydrogenase synthesis (GDH) were about 9 to 12% and 7 to 9% higher in the *OsATG8b*-overexpressing lines under NS and NL conditions, respectively. However, the *osatg8b* mutants showed significant decreases in activities of these N metabolism-related enzymes (decreased by about 7–15% in NS and 8–23% in NL for both traits) (Fig 3D–3F). Consequently, we concluded that *OsATG8b* plays a positive role in both photosynthesis and N metabolism.

### Enhanced autophagy significantly improved grain yield

**Fig 4:**
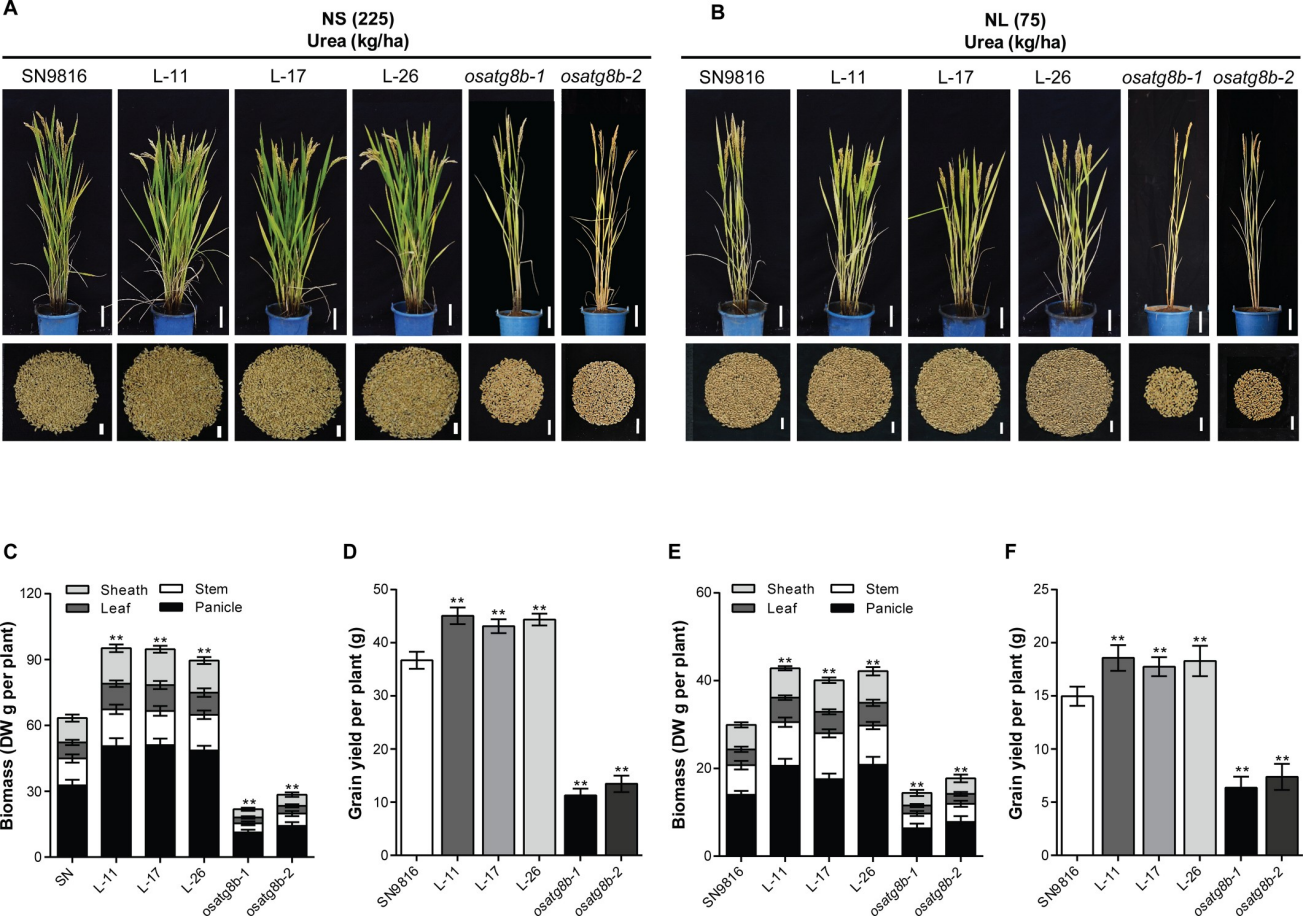
Phenotypes, biomass, and grain yield of rice with altered expression of *OsATG8b*. (A) and (B) Phenotypes of the whole rice plants and total grains per plant in SN9816, *OsATG8b*-overexpressing lines, and *osatg8b* mutants under NS (225 kg·ha^-1^) and NL (75 kg·ha^-1^) at ripening stage. Scale bars, 10 cm and 2 cm. (C) and (D) Biomass and grain yield under NS (225 kg·ha^-1^) condition. (E) and (F) Biomass and grain yield under NL (75 kg·ha^-1^) condition. Values are means ± SD (n = 12), **P < 0.01 (*t*-test).

As expected, lines overexpressing *OsATG8b* exhibited more robust and vigorous growth continued until the ripening stage both under two N conditions (Fig 4A and 4B). Besides, the *OsATG8b*-overexpressing lines had a significantly higher dry weight of panicle, sheath, stem, and leaf under both NS and NL conditions, resulting in about 46.98% and 39.32% increases in biomass, respectively (Fig 4C and 4E). On the contrary, the *osatg8b* mutants displayed inhibitory growth and the biomass accumulation decreased by more than 50% than that of SN9816 under both two N conditions. The potential effect of *OsATG8b* on rice grain yield was further analyzed under both two N conditions. Compared with SN9816, plants overexpressing *OsATG8b* showed significantly increased total grains number, whereas down-regulation of *OsATG8b* induced the opposite effects (Fig 4A and 4B). Further analyzed the major agronomic traits showed that the 1000-grain weight, the number of grains, and second branches per panicle were significantly higher in *OsATG8b*-overexpressing lines under both NS and NL conditions (S2 Table). Although the seed setting rate in the *OsATG8b*-overexpressing lines was decreased relative to SN9816, the actual grain number per plant was still increased by 39.59% and 30.69% under NS and NL conditions, respectively (S2 Table). Thus, the grain yield per plant in the *OsATG8b*-overexpressing lines increased by an average of 20.41% and 21.55% under NS and NL conditions. However, the *osatg8b* mutants displayed dramatically decreases in grain number per panicle and panicles number per plant, resulting in 60.96% and 54.14% reduction in grain yield per plant under NS and NL conditions (Fig 4D and 4F; S2 Table). Taken together, these results suggested that the expression level of *OsATG8b* was directly and positively correlated to the biomass and grain yield.

### Elevated *OsATG8b* gene expression improved N utilization and remobilization efficiency in rice

**Fig 5:**
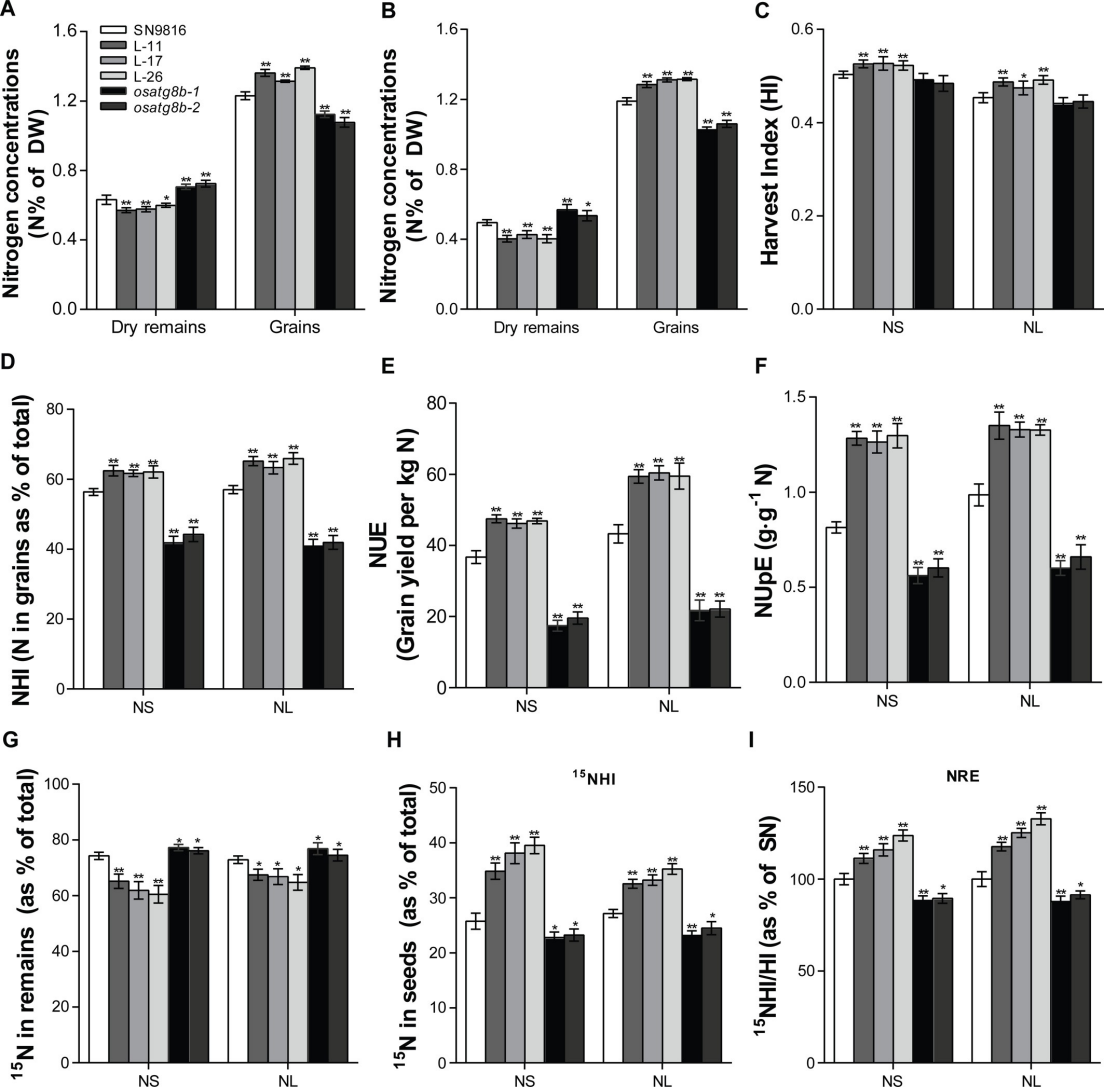
Evaluation of N Utilization Efficiency (NUE) and N Remobilization Efficiency (NRE) in transgenic rice. (A) and (B) N concentration of dry remains (N %_Dr_) and seeds (N %_SEEDS_) under NS (225 kg·ha^-1^) and NL (75 kg·ha^-1^) conditions in SN9816, *OsATG8b*-overexpressing lines and *osatg8b* mutant. (C) Harvest index (HI), (D) Nitrogen harvest index (NHI), (E) Nitrogen use efficiency (NUE), (F) Nitrogen uptake efficiency (NUpE), (G) Partitioning of ^15^N in remains, (H) Partitioning of ^15^N in seeds (^15^NHI) and (I) ^15^NHI/HI ration was calculated as a percentage of SN9816 and used to estimate nitrogen remobilization efficiency (NRE) in SN9816 and transgenic rice under NS (225 kg·ha^-1^) and NL (75 kg·ha^-1^) conditions. Values are means ± SD (n = 12), *P < 0.05, **P < 0.01 (*t*-test).

Given the increased yield and biomass in *OsATG8b*-overexpressing lines, we next assessed whether altered the expression level *OsATG8b* affected N utilization. Compared with SN9816, the lines overexpressing *OsATG8b* accumulated more N in seeds but had lower N in dry remains, which reduced the waste of N under both NS (225 kg·ha^-1^) and NL (75 kg·ha^-1^) conditions. By contrast, N accumulation in *osatg8b* mutants was significantly decreased in seeds but increased in dry remains (Fig 5A and 5B). In accordance with the higher yield and N content of seeds from *OsATG8b*-overexpressing lines, the HI and NHI were significantly higher in the transgenic lines than in SN9816 both under NS (increased by 4.38% and 10.16%) and NL (increased by 6.79% and 13.64%) conditions (Fig 5C and 5D). However, *osatg8b* mutants showed a little but not significant decrease in HI and a significant decrease in NHI (decreased by 21.87% in NS and 28.11% in NL). As expected, the NUE of *OsATG8b*-overexpressing lines was significantly increased by 27.51% relative to SN9816 under the NS condition but decreased by 52.55% in *osatg8b* mutants (Fig 5E). More excitingly, the NUE of *OsATG8b*-overexpressing lines were 38.16% higher compared with SN9816 under the NL condition, while the *osatg8b* mutants exhibited a 49.76% reduction in NUE (Fig 5E). Consistent with NUE, NUpE was also significantly increased in the *OsATG8b*-overexpressing lines, but greatly repressed in *osatg8b* mutants both under two N conditions (Fig 5F). These results suggested that *OsATG8b* confers a substantial improvement in NUE and NUpE, which was particularly useful for the maintenance of plant fitness and adaptation to nutrient limitation.

To accurately monitor the movement of N, the partitioning of ^15^N in SN9816 and transgenic lines was tracked. This analysis showed that overexpression of *OsATG8b* accumulated more ^15^N in seeds (increased by 45.6% in NS and 24.01% in NL), and reduced the partitioning of ^15^N into the remains relative to SN9816 under both N nutrient solutions (decreased by 15.81% in NS and 8.95% in NL). Whereas the opposite results were found in *osatg8b* mutants (Fig 5G and 5H). The ratio of ^15^NHI to HI was next used to compare the NRE as described previously [5, 21]. Compared to SN9816, the ^15^NHI/HI ratio was 16.96% and 25.19% higher in *OsATG8b*-overexpressing lines than in SN9816 under NS and NL conditions, while decreased by 11.71% and 12.22% in *osatg8b* mutants (Fig 5I). These results suggested that overexpression of *OsATG8b* significantly improved N remobilization into seeds, which resulted in favorably increased yield and NUE under varying N conditions.

### Transcriptional profiling of rice with enhanced autophagy

**Fig 6:**
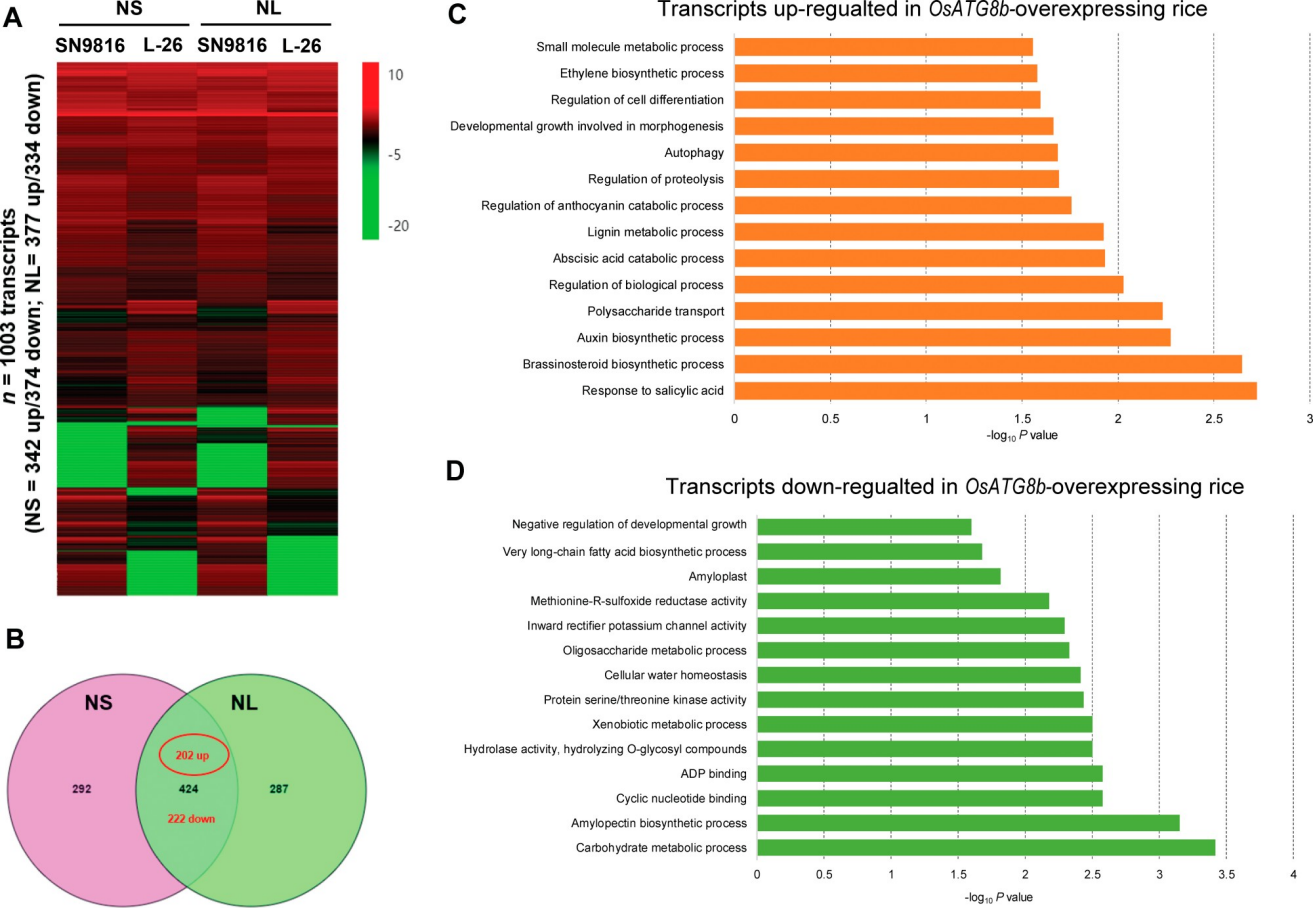
Transcriptional trends characteristic for *OsATG8b*-overexpressing rice under NS (225 kg·ha^-1^) and NL (75 kg·ha^-1^) conditions at the grain-filling stage. (A) Heat maps showing the transcripts differentially expressed in *OsATG8b*-overexpressing rice versus SN9816 under NS and NL conditions. The color legend indicates normalized gene expression values. (B) Venn diagram showing the overlap in the numbers of transcripts that were consistently up-or down-regulated in *OsATG8b*-overexpressing rice versus SN9816 grown under NS and NL conditions. (C) and (D) Gene ontology enrichment of DEGs “up-regulated” and “down-regulated” in *OsATG8b*-overexpressing rice versus SN9816 both under NS and NL conditions.

To investigate the possible molecular mechanism by which *OsATG8b* overexpression affects rice growth and yield, differences between the global gene expression of SN9816 and *OsATG8b*-overexpressing rice were determined by RNA-sequencing. We identified 1,003 DEGs by comparing the gene expression levels in *OsATG8b*-overexpressing rice to those in SN9816 under NS or NL conditions (Fig 6A). Of a core set of 424 genes that were significantly differentially expressed, 202 were up-regulated and 222 were down-regulated (Fig 6B). GO enrichment analysis (*P* < 0.05) showed that genes involved in processes such as biological process regulation, cell differentiation regulation, and plant hormones were up-regulated in *OsATG8b*-overexpressing plants (Fig 6C). Among these processes, salicylic acid response, abscisic acid, anthocyanin catabolic process, ethylene biosynthetic process, and polysaccharide transport, were previously identified as being impacted by autophagy [46–48]. Conversely, Gene Ontology (GO) enrichment analysis of DEGs down-regulated in *OsATG8b*-overexpressing plants under NS and NL revealed that these genes were related to negative regulation of developmental growth, carbohydrate and oligosaccharide metabolic processes, biosynthesis of amylopectin, cyclic nucleotide and ADP binding, long-chain fatty acid, and hydrolase activity (Fig 6D). Although N metabolism was not identified as a significant GO term, real-time RT-PCR revealed that the transcript levels of several genes known to be involved in N metabolism [17, 49–52] were up-regulated under both NS and NL conditions (S3 Fig). As shown in S3 Table, the impact of *OsATG8b* overexpression on transcript abundance was confirmed for several selected DEGs. These results indicate that *OsATG8b* profoundly influences the rice transcriptome and has great potential for improving growth and development.

### *OsATG8b* overexpression conferred high yield and NUE to rice under different genetic backgrounds

**Fig 7:**
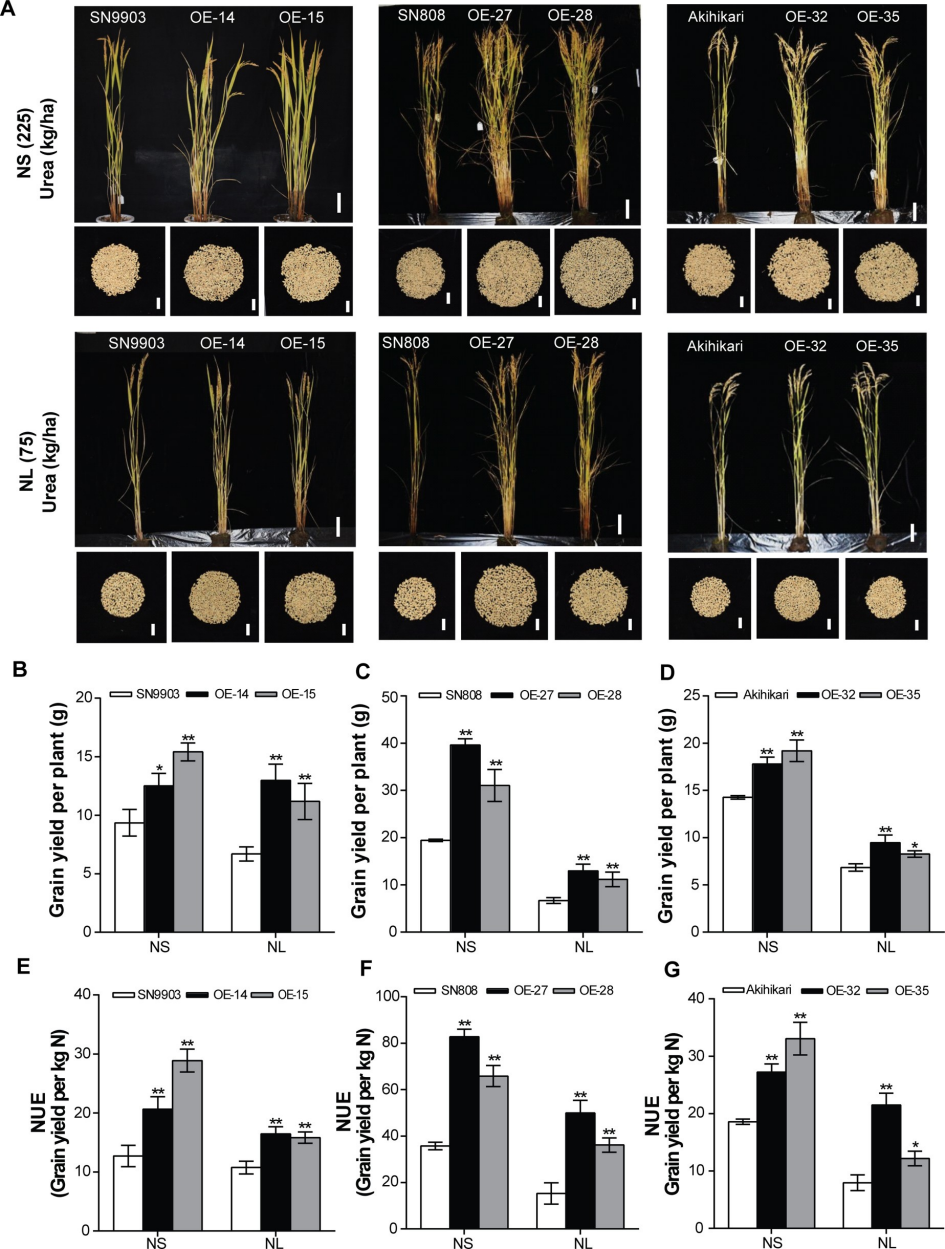
Overexpression of *OsATG8b* increased grain yield and NUE in rice under different genetic backgrounds. (A) Phenotypes of the whole rice plants and total grains per plant in SN9903, SN808, Akihikari, and their *OsATG8b*-overexpressing lines under NS (225 kg·ha^-1^) and NL (75 kg·ha^-1^) at ripening stage. Scale bars, 10 cm and 2 cm. (B)-(D) Grain yield of *OsATG8b*-overexpressing lines in different genetic backgrounds under NS (225 kg·ha^-1^) and NL (75 kg·ha^-1^) conditions. (E)-(G) Nitrogen use efficiency (NUE) of *OsATG8b*-overexpressing lines in different genetic backgrounds under NS (225 kg·ha^-1^) and NL (75 kg·ha^-1^) conditions. Values are means ± SD (n = 12), *P < 0.05, **P < 0.01 (*t*-test).

To further verify whether overexpression of *OsATG8b* in other rice cultivars would similarly be boosted in yield and NUE, we generated *OsATG8b*-overexpressing transgenic rice in other *japonica* cultivars, including Shennong9903 (SN9903), Shennong808 (SN808) and AKihiRari. All the transgenic plants displayed similar phenotypes with the SN9816 background's *OsATG8b*-overexpressing lines, which produced more grains compared to their wild types both under NS and NL conditions (Fig 7A). In accordance with the improved growth phenotypes, the grain yield in these overexpressing lines was also significantly increased both under NS and NL conditions (Fig 7B–7D). Besides, the NUE of these transgenic lines in different backgrounds were also greatly increased under two N conditions (Fig 7E–7G). Taken together, the high yield and NUE of *OsATG8b*-overexpressing transgenic rice under different backgrounds, indicating the functions of *OsATG8b* in rice are stable and heritable.

## Discussion

In plants, autophagy is essential for proper nutrient allocation, although it operates at a low constitutive level [42, 53, 54]. Autophagy has two opposite roles during leaf senescence and nutrients starvation: one is to maintain longevity through the circulation of nutrients; the other is to participate in the systematic degradation of tissues and export of recyclable nutrients to areas of growth and storage [19]. Because of these roles, autophagy can be used to regulate important agricultural traits, tolerance to abiotic and biotic stress, and mitigate the effects of nutrient depletion. Consistent with the high autophagy activity, the transcription of many plant *ATG* genes is significantly up-regulated under nutrient-limited conditions [4, 8, 13, 55]. In agreement with previous studies, we confirmed that the transcript level of rice *OsATG8b* and the number of YFP-OsATG8b labeled autophagosomes were significantly increased under N deficiency (Fig 1B), indicating that *OsATG8b*-mediated autophagy may confer tolerance to N stress in rice. Minina et al. mentioned that overexpression of *ATG5* or *ATG7* in Arabidopsis increased autophagic activity [43]. Here, we confirmed that elevated autophagic activity due to overexpression of *OsATG8b* could significantly increase the tolerance to N starvation, which was presumably associated with the increased formation and triggering of autophagosomes to degrade the insoluble proteins.

N availability strongly influences grain yield because N utilization is tightly associated with the plant’s life cycle, and there are sophisticated mechanisms for N uptake, translocation, assimilation, and remobilization [17, 56]. Autophagy plays a vital role in N management in plants when N starvation. When autophagy is defective, redundant N in the cells cannot be effectively recovered or utilized, leading to nutrient accumulation, wastage, cell death, and lower amino acid content, all of which inhibit plant growth and reduce NUE [57]. Thus, modifying the autophagy process in crops could improve NUE and increase crop yield [58]. Our results support this conclusion and indicate that *OsATG8b* plays a critical role in N uptake and utilization. Functional characterization of the *OsATG8b* loss-of-function mutants and overexpressed transgenic lines demonstrated that overexpression of *OsATG8b* could increase the activity and expression of key enzymes and genes related to N metabolism (Fig 3D–3F and S3 Fig), which promoted the N uptake and assimilation in rice. Thus, the *OsATG8b*-overexpressing lines had significantly higher NHI and NUpE, especially NUE, and the opposite results in *osatg8b* mutant under both NS and NL conditions (Fig 5D–5F). Since lipidation of OsATG8b was greatly increased in *OsATG8b*-overexpressing lines but decreased in the *osatg8b* mutant, we considered that all of these presumably because of the increased autophagy flux.

Another significant finding of this study was that overexpression of *OsATG8b* could enhance the reuse and remobilization of N from the vegetative tissues, increase the transfer of ^15^N to seeds, which causes increased NRE in rice (Fig 5G–5I). As 75% to 80% of N in the leaf is present in the chloroplast, and the main forms are Rubisco and chlorophyll [59], the higher chlorophyll content and Rubisco activity during the grain-filling stage conferred a higher capacity to convert light energy into photosynthetic products. Besides, the increased autophagic activity in the *OsATG8b*-overexpressing lines resulted in an accelerated overall of the N metabolic cycle, so more N (nutrients) could be mobilized to chloroplasts to produce organic materials. Consequently, the *OsATG8b*-overexpressing lines showed obviously increased biomass and grain yield. N partitioning studies in maize *atg12* mutants implied that the reduced seed yield presumably because of impaired N recycling by autophagy [5]. Our studies also indicated that the greatly increased grain yield in *OsATG8b*-overexpressing lines was at least partially caused by the enhanced autophagic recycling leading to efficient N cycling from the leaves to the reproductive organ (seeds), which reduced the waste of available N and promoted the utilization and remobilization of N. Additionally, the increased number of the effective panicles number and secondary branches leading to significant increases in total grains number per plant, as well as the higher 1000-grain weight (S2 Table), could also explain the high productivity in *OsATG8b*-overexpressing lines. Excitingly, the *OsATG8b*-overexpressing transgenic rice under different backgrounds consistently displayed markedly increased NUE and grain yield (Fig 7), further confirming the great potential of *OsATG8b* for yield improvement in rice.

Consistent with the obvious improvement of the phenotype in *OsATG8b*-overexpressing lines, elevated *OsATG8b* expression broadly impacted the transcriptomes of rice. Our RNA-seq analyses showed that the affected transcripts trend of SN9816 in vivo was similar in the heat map both under NS and NL conditions, so did the transgenic lines (Fig 6A), suggesting that the ability to resist low N stress in the transgenic rice was due to the overexpression of *OsATG8b*. Previous metabolomics studies reported that the autophagy-deficient mutants might have increased synthesis of the stress hormones salicylic acid and abscisic acid, which could be connected to the hyperaccumulation of flavonoid or anthocyanin in *atg* mutants [21, 46, 47]. Similarly, our results showed that overexpression of *OsATG8b* significantly up-regulated genes involved in the response to salicylic acid, synthesis of growth-promoting hormones (auxin and brassinosteroids), biological regulation, cell differentiation, and morphogenesis, etc. Brassinosteroids and auxin have a synergistic effect on plant development by regulating cell elongation, cell division, and cell differentiation [60, 61]. Moreover, recent research in tomato showed that brassinosteroids-induced autophagy involved in N remobilization and mediated the response to N starvation [62]. It might partially explain the remarkably increased biomass and seed production as well as the improved NRE and resistance to N starvation in *OsATG8b*-overexpressing rice. In addition, the up-regulation of genes involved in autophagy regulation, the proteasome, and the small molecule metabolic process (Fig 6C) further confirm that *OsATG8b* has a stimulatory effect on autophagic activity and that its overexpression increases the clearing and recycling of unwanted or dysfunctional complexes. Studies in Arabidopsis showed that the transport of sugars from rosette leaves to the inflorescence plays a vital role in sustaining seed development [63], and higher seed yield in *ATG*-overexpressing plants might be attributed to up-regulation of genes involved in sugar transport [43]. Our data showed that overexpression of *OsATG8b* significantly up-regulated genes involved in polysaccharide transport (Fig 6C), which could also partially explain the improved N remobilization and yield of transgenic rice. By contrast, genes involved in carbohydrate and oligosaccharide metabolic processes, biosynthesis of amylopectin and long-chain fatty acids, and hydrolase activity were significantly down-regulated in transgenic rice (Fig 6D), possibly indicating that overexpression of *OsATG8b* could effectively promote the autophagy recycling pathway during plant growth and grain formation and consequently reduce the demand for several biosynthesis and metabolic pathways. Besides, overexpression of *OsATG8b* broadly and directly impacted the expression of plant metabolism genes. This result suggested that the cellular metabolic status in transgenic rice might be altered by regulating enzyme activity in several processes, which made a benefit to push the development in an irreversible direction and to recycle the nutrient sequentially.

The *OsATG8a*, *OsATG8b*, and *OsATG8c* belong to the same family and they maybe have redundant functions in rice. In our previous studies, it was found that the expressions of other *OsATG8*s were up-regulated during N-limited conditions, and overexpressed *OsATG8a* or *OsATG8c* in transgenic rice could increase autophagy activity and confer tolerance to N limitation [40, 41]. Besides, our group was the first to show that overexpression of *OsATG8b* could increase the autophagy activity and NRE in transgenic Arabidopsis, leading to significantly higher NUE and yield [64]. This research further confirmed that overexpression of *OsATG8b* has enormous potential as a strategy to increase yields and NUE in both monocot and eudicot crops. A recent research by Fan et al. showed that *OsATG8b*-mediated autophagy is involved in N recycling to grains and contributes to the grain quality in rice under normal growth conditions [65]. We two did completely independent research and got similar conclusions during the same period, which should be the verification and support for each other. However, there were also clear differences between us. In Fan’s study, the *OsATG8b*-RNAi and *OsATG8b*-OE lines showed a relatively normal phenotype under both low N and high N levels at the vegetative stages, and they considered that *OsATG8*s function redundantly in response to nutrient stress [65]. In our research, the *OsATG8b*-overexpressing lines performed better than the control group at all growth stages, especially under the NL condition, while the *osatg8b* mutants showed severe growth retardation under both two N conditions. Another point worth mentioned was that the NL treatment that we used (one-third of the N in the NS treatment) was already a very harsh condition. Even under this harsh condition, the *OsATG8b*-overexpressing lines could still use the restricted amount of N transferred to the seeds more effectively, enhance internal N remobilization, and improved yield and NUE more efficiently. In addition, compared the observed phenotypes and autophagic activity of three *OsATG8*s-related overexpression transgenic rice and the mutants, *OsATG8b* was the most significant and obvious, followed by *OsATG8a*, while *OsATG8c* was the weakest. Intriguingly, it was found that *osatg8b* knockout mutants caused downregulation of the *OsATG8a* and *OsATG8c* although their coding sequences were not changed. Furthermore, there were no such remarkable phenotypes as *osatg8b* mutants had been observed in the *osatg8a* and *osatg8c* mutants. We considered that this might be due to the high levels of amino acid sequence identity between these 3 genes, and *OsATG8b* was the dominant gene of *ATG8*s in rice. The severe reduction of NUE, NRE, and yield in *osatg8b* mutants further supports this conclusion.

Collectively, our results show that elevated *OsATG8b* expression can increase autophagy activity, photosynthetic products, NUE, and grain yield. Additionally, *OsATG8b*-mediated increased autophagy can effectively regulate N remobilization and plays a positive role in the maintenance of plant fitness and adaptation to nutrient limitation. Our results revealed a new mechanism by which autophagy increases NUE and yield in rice, it will greatly help in exploring the application value of autophagy and provide a beneficial strategy to increase NUE and crop yield through manipulation of autophagy and N recycling, especially under suboptimal growth conditions. Impressively, the effect of *OsATG8b* was not only verified in different rice backgrounds but also in Arabidopsis, indicating the great potential for the use of this gene in crop improvement programs for multiple crop species.

## Materials and methods

### Plant materials and growth conditions

Rice (*Oryza sativa*, *japonica*) varieties SN9816, SN9903, SN808, and Akihikari were used as the wild type and recipient for genetic transformation in this study. For N-limitation hydroponic culture experiment, rice seedlings were cultured with modified International Rice Research Institute (IRRI) nutrient solution (pH = 5.7) as described previously [66] with different concentrations of N in an artificial climate growth chamber as described. The sufficient-N nutrient solution contained the following macronutrients (mM): (NH_4_)_2_SO_4_ (0.72), Ca(NO_3_)_2_·4H_2_O (0.72), CaCl_2_ (0.28), K_2_SO_4_ (0.36), KH_2_PO_4_ (0.32), MgSO_4_·7H_2_O (1.67), Na_2_SiO_3_·9H_2_O (1.60) and micronutrients (μM): MnCl_2_·4H_2_O (9.11), (NH_4_)_6_Mo_7_O_24_·4H_2_O (0.074), H_3_BO_3_ (18.52), ZnSO_4_·7H_2_O (0.15), CuSO_4_·5H_2_O (0.16), Fe(II)-EDTA (35.81). The modified IRRI nutrient solution with 0.24 mM (NH_4_)_2_SO_4_ and 0.24 mM Ca(NO_3_)_2_·4H_2_O was used for low-N (NL), and the N-free solution for deficient-N (ND), the lack of Ca^2+^ was replaced with CaCl_2_.

### Binary vector construction and rice transformation

The *OsATG8b* gene has that encodes 120 amino acids. To construct *OsATG8b*-overexpressing lines, a 360-bp coding sequence (CDS) of *OsATG8b* (*LOC_Os04g53240*; *Os04g0624000*) was amplified by PCR from cDNA of SN9816 and ligated into binary vector pCAMBIA1301. The *35S-YFP-OsATG8b* fusion construct was produced by inserting the complete coding region of *OsATG8b* into the pCAMBIA1300-35S-YFP vector. The *OsATG8b* CRISPR vector construct was prepared using a plant CRISPR/Cas9-based genome editing system as described [67]. We designed sgRNA to target specific sites in the first exon of *OsATG8b* by using E-CRISPR (http://www.e-crisp.org/E-CRISP/index.html), and a pair of DNA oligonucleotides with appropriate cloning linkers were synthesized for the target site, then annealed and ligated into *Bsa* I-digested pRGE32 vectors. All the constructions were confirmed by DNA sequencing. The rice transformation was conducted as described previously [68]. The *OsATG8b*-overexpressing and *35S-YFP-OsATG8b* transgenic lines were screened by PCR amplification with the hygromycin gene, the *osatg8b* mutants were screened by DNA sequencing with specific primers, and the T_3_ homozygous transgenic plants were taken for the experiments. The corresponding primers used in this study were listed in S1 Table.

### Total RNA extraction and gene expression analysis

Total RNA extraction was conducted using the Eastep Super Total RNA Extraction Kit (Promega) and the first-strand cDNA was synthesized with the PrimeScript RT Master Mix (TaKaRa). Real-time RT-PCR was performed as described previously [40] by using SYBR Premix Ex TaqII (TaKaRa) on an Applied Biosystems 7500 Real-Time PCR System. The following standard thermal profile was used for all PCRs: 95°C for 30 s, 40 cycles of 95°C for 5 s, and 60°C for 34 s. All reactions were done at least in triplicates. *OsActin1* was used as an internal control. The primers used for RT-PCR analysis were listed in S1 Table. Three independent biological replicates were conducted.

### Protein isolation and western blot analysis

14-day-old seedlings of SN9816, *OsATG8b*-overexpressing lines and *osatg8b* mutants cultured hydroponically with NS solution were transferred to NS and ND solution with DMSO or 0.5 μM ConA (Sigma-Aldrich) dissolved in DMSO with or without 5 μM wortmannin (Sigma-Aldrich) for 24h. The full-length OsATG8b was used to raise a polyclonal antibody (GenScript, Nanjing China). Anti-AtNBR1 and Anti-Tubulin (Agrisera) were used as primary antibodies (1: 1000), goat anti-rabbit (IgG) (Sigma-Aldrich) was used as the secondary antibody (1: 10000). Total protein extraction and membrane protein extraction were conducted using the Minute™ Total Protein Extraction Kit and Membrane Protein Isolation Kit for plants (Invent Biotechnologies, Inc), then the protein concentration was determined with the BCA Protein Assay Kit (TaKaRa) referred to the instructions. Immunoblotting was done as described [40]. The obtained proteins were loaded into a 12–15% SDS-PAGE with 6 M urea gel. After electrophoresis separation, the proteins were transferred to a PVDF membrane for protein profiles analysis or immunoblot analysis. Three independent biological replicates were conducted.

### Confocal microscopy for detecting autophagosome activity

14-day-old seedlings of *35-YFP-OsATG8b* transgenic rice seedlings were transferred to NS (2.88 mM N) or ND (0 mM N) liquid medium with or without 0.5 μM ConA for 14h, and root epidermal cells of the elongation zone were observed by confocal microscope (Zeiss LSM 780) as described [39]. The YFP-OsATG8b labeled autophagosomal structures were monitored by a 514 nm excitation laser. The average number of puncta for three cells belonging to the same root was considered as a single measurement, at least 10 seedlings per genotype per treatment were imaged and then counted by Image J.

### Field trials of rice

Field tests were performed under natural growth conditions from 2016 to 2019 during the rice-growing season at the experimental farm of Shenyang Agricultural University (Shenyang, China; Longitude: 123.34°E Latitude: 41.49°N). The brown loam soil used in our experiment contained organic matter of 16.3 g·kg^-1^, total N of 0.88 g·kg^-1^, available P of 28.64 mg·kg^-1^, available K of 114.04 mg·kg^-1^, and pH = 7.11. Rice plants were cultivated in pots, each pot containing four plants and ten pots as replicates were planted for each N condition or genotype. There were three N levels in this experiment NS (225 kg·ha^-1^), NL (75 kg·ha^-1^), and ND (without N fertilizer) as blank control. Urea was used as the only N source for NS and NL conditions and applied for three times as base, tillering and panicle fertilization, with 36%, 24%, and 40% of total applied urea respectively. 112.5 kg·ha^-1^ P_2_O_5_ was supplied as basic fertilizer, and 112.5 kg·ha^-1^ K_2_O was supplied as basic fertilizer and panicle fertilizer twice. Superphosphate (12% P_2_O_5_) and potassium chloride (60% K_2_O) were used as phosphate and potassium fertilizers.

### Measurement of total chlorophyll, leaf enzyme activity, and photosynthetic characters

Flag leaves at the grain-filling stage (18 days after heading) were sampled, and 12 plants for each genotype under NS (225 kg·ha^-1^) or NL (75 kg·ha^-1^) conditions were randomly selected as replicates to measure. Total chlorophyll content was measured as described [69], about 0.5 g flag leaves were collected from the main stem in 10 mL 96% ethanol, and total chlorophyll content was calculated spectrophotometrically based on the absorbance of the supernatant at 649 and 665 nm. The activities of nitrate reductase (NR, EC 1.6.6.2), glutamine synthetase (GS, EC 6.3.1.2), and glutamate dehydrogenase synthesis (GDH, EC 1.4.1.2) were determined using the enzyme activity assay kits (Shanghai Enzyme-linked Biotechnology Co., Ltd., China), about 0.5 g flag leaves were collected from the main stem and frozen in liquid N, and then stored at -80°C until measurements. The procedures were conducted following the instructions provided by the manufacturer. A portable infrared gas analyzer (CIRAS 3, PP Systems, Amesbury, MA, USA) was used to measure net photosynthetic rate (*P*_N_) at the heading stage on flag leaves during 9:00 am-12:00 pm on sunny days. The approximate sunrise time for the period in which the photosynthetic measurements was 4:45 am. Photosynthetic photon flux densities of 1200 μmol m^-2^s^-1^) were provided by a LED source, CO_2_ concentration was provided by the CO_2_ injector system and maintained at 390 ppm in the leaf chamber.

### Biomass, total N content, NUpE, NUE calculations, and agronomic traits analysis

Rice plants grown under NS (225 kg·ha^-1^), NL (75 kg·ha^-1^) and ND (without N fertilizer) conditions were sampled at the ripening stage by separation into the stem, leaf sheath, leaf blade and panicle at harvest, then all samples were kept in a dry oven at 80°C for 7 days, and dry mass was weighted as DW_Dr_ (dry weight of stem, leaf sheath, and leaf blade) and DW_seeds_ (dry weight of total seeds). Then the dry mass of all these tissue organs was ground to powder, total N concentrations (N%) were measured using an Elemental Analyzer (Elementa, Vario MAX C, Germany), which presented as N% = mg N / 100 mg DW. Biomass was calculated as DW_Dr_ + DW_Seeds_. The harvest index (HI) was calculated as DW_Seeds_ / (DW_Seeds_ + DW_Dr_) ratio and was used as an important indicator for yield. The NHI was calculated as (N%_Seeds_ × DW_Seeds_) / (N%_Seeds_ × DW_Seeds_ + N%_Dr_ × DW_Dr_). The NUpE was calculated as (total aboveground N accumulation in NS or NL pot)–(total aboveground N accumulation in ND pot) / N supply, and the NUE was calculated as (grain yield in NS or NL pot)–(grain yield in ND pot) / N supply. The agronomic traits including the seed-setting rate, thousand-grain weight, panicles number, and grain yield were analyzed at the ripening stage according to the methods for measurement described previously [49]. The panicle branches are lateral organs at the reproductive stage of rice, which are composed of primary branches and secondary branches. All the above traits were measured on a single-plant basis, and 12 plants under each N conditions were randomly selected as replicates to measure.

### ^15^N-Labeling and NRE calculations

SN9816, *OsATG8b*-overexpressing lines, and *osatg8b* mutants were grown hydroponically with NS or NL nutrient solution as above. The ^15^N-Labeling experiment was conducted as previous studies described [5, 14] at the booting stage (70 days after sowing) when most of the 11th leaves on the main stem just emerged from the sheath of the 10th leaves, the nutrient solution was replaced with the ^15^N-labeled (^15^NH_4_)_2_SO_4_ and Ca(^15^NO_3_)_2_·4H_2_O (10.6 atom% excess) as ^15^NS or ^15^NL nutrient solution for 3 d. Afterward, the pots were applied with NS or NL solution every 3 days until plant maturity, then plants were separated into seed, stem, leaf sheath, and leaf blade. The ^15^N abundance, ^15^NHI, and NRE were estimated as described [20]. All the above traits were measured on a single-plant basis, and 12 plants under each N conditions were randomly selected as replicates to measure.

### RNA-sequencing analysis

Flag leaves of SN9816 and *OsATG8b*-overexpressing rice under NS and NL growth conditions were sampled at the grain-filling stage. For each N treatment, three independent biological replicates were conducted. Sequencing libraries were generated using NEBNext UltraTM RNA Library Prep Kit for Illumina (NEB, USA) following the manufacturer’s recommendations and index codes were added to attribute sequences to each sample. RNA quality was determined using Agilent 2100 Bioanalyzer (Agilent Technologies). RNA libraries were constructed from 1 μg of total RNA and subjected to deep sequencing at an Illumina Hiseq 2500 platform (BioMarker Technologies). Differentially expressed genes (DEGs) were identified by DEseq2 with the criteria of absolute log2 (fold change) ≥1 and false discovery rate (FDR) ≤ 0.05. GO enrichment analysis of the differentially expressed genes (DEGs) was implemented by the GOseq R packages based Wallenius non-central hypergeometric distribution [70].

### Statistical analysis

The statistically significant differences of all data were analyzed based on Student's *t*-test with the comparisons between two groups of data separately (*OsATG8b*-overexpressing lines vs SN9816 and mutants vs SN9816) at significance levels of *P < 0.05 and **P < 0.01.

## Supporting information

S1 Fig Real-time RT-PCR analysis of the transcript levels of *OsATG8*s in 14-day-old seedlings of SN9816, *OsATG8b*-overexpressing lines, and *osatg8b* mutants. *OsActin1* was used as an internal control. Values are means ± SD (n = 3), **P < 0.01 (*t*-test).
(DOCX)

S2 Fig The anti-OsATG8b antibody could not distinguish OsATG8a, OsATG8b, and OsATG8c proteins. Immunoblot analysis of the protein level of The OsATG8a, OsATG8b, or OsATG8c expressed in *E*. *coli* with the anti-OsATG8b.
(DOCX)

S3 Fig Overexpression of *OsATG8b* affected the expression of marker genes in N metabolism. qRT-PCR validation of several known genes related to N metabolism in flag leaves of SN9816 and *OsATG8b*-overexpressing rice under NS (225 kg·ha^-1^) and NL (75 kg·ha^-1^) conditions at grain-filling stage. *OsActin1* was used as an internal control. Values are means ± SD (n = 3), *P < 0.05, **P < 0.01 (*t*-test).
(DOCX)

S1 Table The information of primers used in this study. (DOCX)

S2 Table Yield-related characteristics of SN9816 and transgenic rice under different N conditions. (DOCX)

S3 Table Confirmation of RNA-Seq expression profiles with qRT-PCR. (DOCX)

S1 Raw images (PDF)
