# Supplementary material for: Autophagy mediates grain yield and nitrogen stress resistance by modulating nitrogen remobilization in rice
Source: PLoS One. 2021 Jan 14;16(1):e0244996. doi: 10.1371/journal.pone.0244996 (PMC7808584; doi:10.1371/journal.pone.0244996)
Supplement: S1 Fig — OsActin1 was used as an internal control. Values are means ± SD (n = 3), **P < 0.01 (t-test). (DOCX) [file pone.0244996.s001.docx]

**S1 Fig. Real-time RT-PCR analysis of the transcript levels of *OsATG8*s in 14-day-old seedlings of SN9816, *OsATG8b*-overexpressing lines, and *osatg8b* mutants.** *OsActin1* was used as an internal control. Values are means ± SD (n=3), ^**^*P* < 0.01 (*t*-test).
