# Supplementary material for: Autophagy mediates grain yield and nitrogen stress resistance by modulating nitrogen remobilization in rice
Source: PLoS One. 2021 Jan 14;16(1):e0244996. doi: 10.1371/journal.pone.0244996 (PMC7808584; doi:10.1371/journal.pone.0244996)
Supplement: S2 Fig — Immunoblot analysis of the protein level of The OsATG8a, OsATG8b, or OsATG8c expressed in E. coli with the anti-OsATG8b. (DOCX) [file pone.0244996.s002.docx]

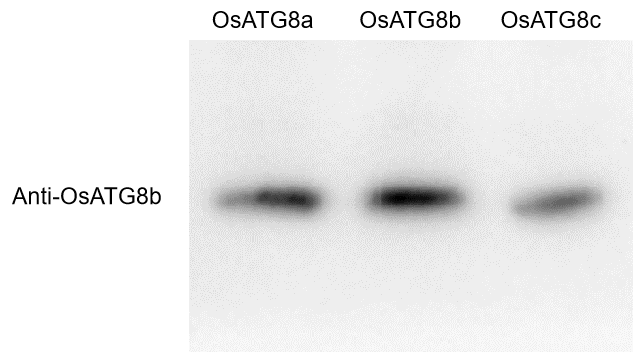


**S2 Fig. The anti-OsATG8b antibody could not distinguish OsATG8a, OsATG8b, and OsATG8c proteins.** Immunoblot analysis of the protein level of The OsATG8a, OsATG8b, or OsATG8c expressed in *E. coli* with the anti-OsATG8b.
