# Supplementary material for: Autophagy mediates grain yield and nitrogen stress resistance by modulating nitrogen remobilization in rice
Source: PLoS One. 2021 Jan 14;16(1):e0244996. doi: 10.1371/journal.pone.0244996 (PMC7808584; doi:10.1371/journal.pone.0244996)
Supplement: S3 Fig — qRT-PCR validation of several known genes related to N metabolism in flag leaves of SN9816 and OsATG8b-overexpressing rice under NS (225 kg·ha-1) and NL (75 kg·ha-1) conditions at grain-filling stage. OsActin1 was used as an internal control. Values are means ± SD (n = 3), *P < 0.05, **P < 0.01 (t-test). (DOCX) [file pone.0244996.s003.docx]

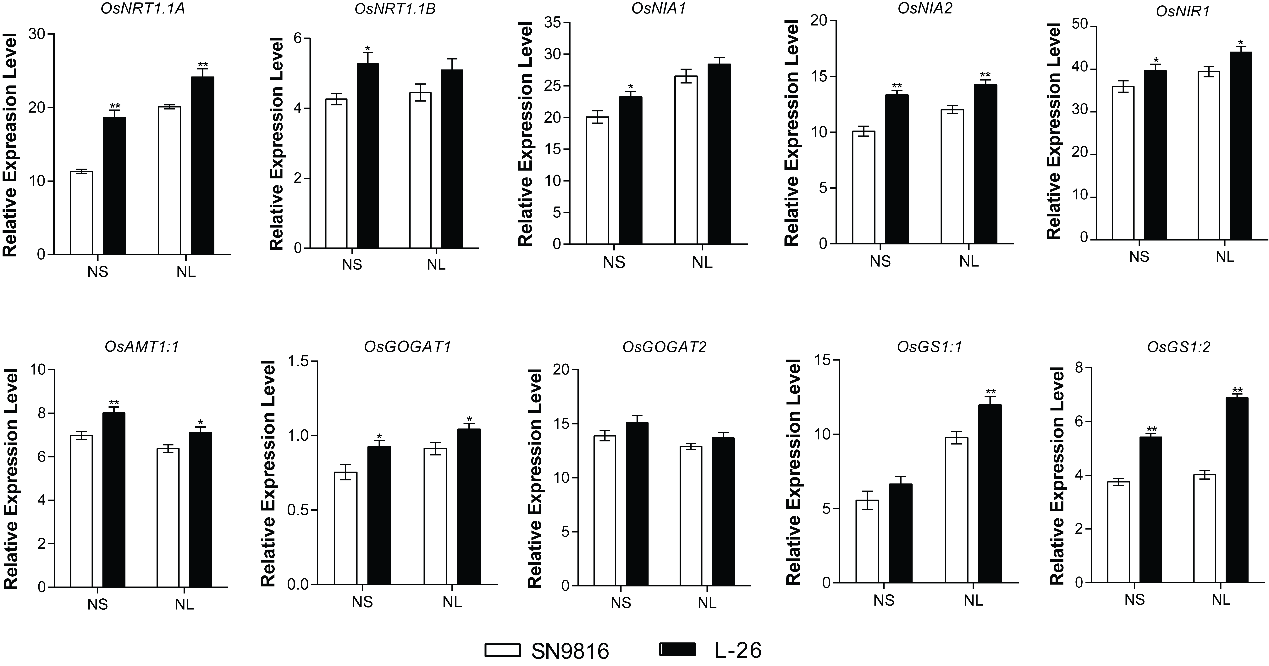


**S3 Fig. Overexpression of *OsATG8b* affected the expression of marker genes in N metabolism.** qRT-PCR validation of several known genes related to N metabolism in flag leaves of SN9816 and *OsATG8b*-overexpressing rice under NS (225 kg·ha^-1^) and NL (75 kg·ha^-1^) conditions at grain-filling stage. *OsActin1* was used as an internal control. Values are means ± SD (n=3), ^*^*P* < 0.05, ^**^*P* < 0.01 (*t*-test).
