# Supplementary material for: Autophagy mediates grain yield and nitrogen stress resistance by modulating nitrogen remobilization in rice
Source: PLoS One. 2021 Jan 14;16(1):e0244996. doi: 10.1371/journal.pone.0244996 (PMC7808584; doi:10.1371/journal.pone.0244996)
Supplement: S1 Table — (DOCX) [file pone.0244996.s004.docx]

| **S1 Table. The information of primers used in this study** | | |
| --- | --- | --- |
| Primer name | Sequence (5´→ 3´) | Functions |
| cOsATG8b-F | CCATTCAAGTGGATGGCCAAGAGCTCGTTCAAGC | Gene cloning |
| cOsATG8b-R | GGTGACCTAGAGCAGCCCAAAGGTGTTCTCG | Gene cloning |
| Osatg8b_sgRNA-_F | GTTGTTGTGTTTCGTTGCGC | CRISPR/Cas9 |
| Osatg8b_sgRNA-_R | AAGCCATCCCCTTAAACCGG | CRISPR/Cas9 |
| rtOsATG8b-F | GAGGCTAACCGTATCAGAGAAA | Real-time RT-PCR |
| rtOsATG8b-R | ACCAGGTACTTTTTCTTGTCGA | Real-time RT-PCR |
| rtOsNRT1.1A-F | CCCACACCAAGCAATTCAGG | Real-time RT-PCR |
| rtOsNRT1.1A-R | GTCTTCACCTCCTCCACGTC | Real-time RT-PCR |
| rtOsNRT1.1B-F | GGCAGGCTCGACTACTTCTA | Real-time RT-PCR |
| rtOsNRT1.1B-R | AGGCGCTTCTCCTTGTAGAC | Real-time RT-PCR |
| rtOsNIA1-F | TCAAGGTGTGGTACGTGGTG | Real-time RT-PCR |
| rtOsNIA1-R | CGAGGTCATAGCCCATCTTC | Real-time RT-PCR |
| rtOsNIA2-F | TGTACCAGGTCATCCAGTCG | Real-time RT-PCR |
| rtOsNIA2-R | CGATGACGTACCACACCTTG | Real-time RT-PCR |
| rtOsNIR1-F | CTGCCTCACCAAGGACAG | Real-time RT-PCR |
| rtOsNIR1-R | TTCCTACTCCTCGTCCTCCT | Real-time RT-PCR |
| rtOsAMT1.1-F | GGTTTCTCTCCCTCTCCGAT | Real-time RT-PCR |
| rtOsAMT1.1-R | CCACCTTCACACCACACATT | Real-time RT-PCR |
| rtOsGOGAT1-F | GTGCAGCCTGTTGCAGCATAAA | Real-time RT-PCR |
| rtOsGOGAT1-R | CGGCATTTCACCATGCAAATC | Real-time RT-PCR |
| rtOsGOGAT2-F | CCTGTCGAAGGATGATGAAGGTGAAACC | Real-time RT-PCR |
| rtOsGOGAT2-R | GCATGGCCCTACTATCTTCGCATCA | Real-time RT-PCR |
| rtOsGS1.1-F | CACCAACAAGAGGCACAATG | Real-time RT-PCR |
| rtOsGS1.1-R | ACTCCCACTGTCCTGGCAT | Real-time RT-PCR |
| rtOsGS1.2-F | TGTTTCTCCTCATCCCTGC | Real-time RT-PCR |
| rtOsGS1.2-F | TCACAGTCCTCGCTTTGC | Real-time RT-PCR |
| rtOsACTIN1-F | ACCATTGGTGCTGAGCGTTT | Real-time RT-PCR |
| rtOsACTIN1-R | CGCAGCTTCCATTCCTATGAA | Real-time RT-PCR |
| LOC_Os02g03640-F | TGGATGAACGAGAAATCGAAGA | Real-time RT-PCR |
| LOC_Os02g03640-R | AACAGCTCTATGTGGGTACTTC | Real-time RT-PCR |
| LOC_Os08g29020-F | GAGAGGAGAAGGAAAACGAGAT | Real-time RT-PCR |
| LOC_Os08g29020-R | CCTGCTTGTATGTCACTCTACA | Real-time RT-PCR |
| LOC_Os04g53240-F | GAGGCTAACCGTATCAGAGAAA | Real-time RT-PCR |
| LOC_Os04g53240-R | ACCAGGTACTTTTTCTTGTCGA | Real-time RT-PCR |
| LOC_Os10g25040-F | ACTCCATTCGTTTCGTATTCCT | Real-time RT-PCR |
| LOC_Os10g25040-R | CACTTTGCAGACATACACTGTG | Real-time RT-PCR |
| LOC_Os11g02520-F | GCCAAAGAAAGTGTTAGCAAGA | Real-time RT-PCR |
| LOC_Os11g02520-R | ATTGGCACACCTATAGTAGCTC | Real-time RT-PCR |
| LOC_Os11g01010-F | TCTTCTGGAGTCTACACGTCTA | Real-time RT-PCR |
| LOC_Os11g01010-R | GTCTTCTTCCTTGATGCGAATC | Real-time RT-PCR |
| LOC_Os01g57082-F | CTTTCACGGTTGTGGTATATGC | Real-time RT-PCR |
| LOC_Os01g57082-R | TTCCTTCAAGGCACAAAATCTG | Real-time RT-PCR |
| LOC_Os02g02120-F | GCCATAAAGCGCTCAAAGATTA | Real-time RT-PCR |
| LOC_Os02g02120-R | CTTCCACTTCTAAGCAACAACC | Real-time RT-PCR |
| LOC_Os02g13430-F | GTCAAAGGTAAGCGTTAAGGTG | Real-time RT-PCR |
| LOC_Os02g13430-R | CATTGTTGTACCGCTTCGATG | Real-time RT-PCR |
| LOC_Os02g32660-F | ATGATCGATGACATTGCATGTG | Real-time RT-PCR |
| LOC_Os02g32660-R | ACGAGTAGAAGCAAACACGTAG | Real-time RT-PCR |
| LOC_Os02g49510-F | CTCATCAAATCTGCCTGTCTTG | Real-time RT-PCR |
| LOC_Os02g49510-R | CGCGTTTTGATCTCTTACTGAG | Real-time RT-PCR |
| LOC_Os02g53180-F | GGAGGAGAAGTTCAAGGAGTTC | Real-time RT-PCR |
| LOC_Os02g53180-R | CAAATTGCTTCATCACCTGCC | Real-time RT-PCR |
| LOC_Os03g62200-F | GGAGGAGAAGTTCAAGGAGTTC | Real-time RT-PCR |
| LOC_Os03g62200-R | CAAATTGCTTCATCACCTGCC | Real-time RT-PCR |
| LOC_Os04g43800-F | GAGATCAACTCCGTCAACGA | Real-time RT-PCR |
| LOC_Os04g43800-R | CCGTTGTTGTAGAAATCGTTCA | Real-time RT-PCR |
| LOC_Os05g01140-F | GCTGTTGTGAGTAGTGATTAGC | Real-time RT-PCR |
| LOC_Os05g01140-R | GCACTCTGCATCATCTATCATAC | Real-time RT-PCR |
| LOC_Os05g11130-F | AAACCCTAGAGTGGACAGACTA | Real-time RT-PCR |
| LOC_Os05g11130-R | TTACTGCCTTCCTCATTATCCC | Real-time RT-PCR |
| LOC_Os12g02330-F | CACCATCAAGTCTCTCAACCTC | Real-time RT-PCR |
| LOC_Os12g02330-R | AGCCAGCATCGATCGATATATC | Real-time RT-PCR |
| LOC_Os12g07210-F | CGTCAAGAACTACACCTACTGA | Real-time RT-PCR |
| LOC_Os12g07210-R | TCTTGTGATCCTAATTCTCGCA | Real-time RT-PCR |
