# Supplementary material for: Autophagy mediates grain yield and nitrogen stress resistance by modulating nitrogen remobilization in rice
Source: PLoS One. 2021 Jan 14;16(1):e0244996. doi: 10.1371/journal.pone.0244996 (PMC7808584; doi:10.1371/journal.pone.0244996)
Supplement: S2 Table — (DOCX) [file pone.0244996.s005.docx]

**S2 Table. Yield-related characteristics of SN9816 and transgenic rice under different N conditions.**

| Treatment | Material | Grain yield per plant (g) | Grain number per panicle | Panicle number per plant | Seed setting rate (%) | 1,000-grain weight (g) | Primary branch | Secondary branch |
| --- | --- | --- | --- | --- | --- | --- | --- | --- |
| NS  (225 kg·ha^-1^) | SN9816 | 31.69 ± 1.57 | 153.60 ± 9.18 | 10.25 ± 0.75 | 91.32 ± 0.94 | 24.00 ± 0.21 | 14.01 ± 1.07 | 27.60 ± 2.29 |
|  | L-11 | 45.08 ± 1.56^**^ | 211.13 ± 3.01^**^ | 12.25 ± 0.62^**^ | 75.16 ± 0.89^**^ | 25.70 ± 0.28^**^ | 16.17 ± 1.17^**^ | 40.67 ± 2.59^**^ |
|  | L-17 | 43.10 ± 1.38^**^ | 219.89 ± 2.84^**^ | 12.58 ± 0.90^**^ | 75.30 ± 0.94^**^ | 25.25 ± 0.18^**^ | 16.60 ± 0.55^**^ | 39.80 ± 3.15^**^ |
|  | L-26 | 44.35 ± 1.12^**^ | 212.22 ± 4.35^**^ | 12.17 ± 0.87^**^ | 75.86 ± 1.02^**^ | 25.38 ± 0.19^**^ | 16.81 ± 1.30^**^ | 40.42 ± 3.13^**^ |
|  | *osatg8b-1* | 11.28 ± 1.29^**^ | 125.11 ± 9.81^**^ | 4.65 ± 0.55^**^ | 88.82 ± 0.75 | 23.44 ± 0.28 | 9.60 ± 1.14^**^ | 13.80 ± 1.30^**^ |
|  | *osatg8b-2* | 13.46 ± 1.54^**^ | 132.45 ± 6.71^**^ | 4.79 ± 0.78^**^ | 89.07 ± 1.16 | 23.82 ± 0.31 | 10.30 ± 1.35^**^ | 14.60 ± 1.50^**^ |
| NL  (75 kg·ha^-1^) |  |  |  |  |  |  |  |  |
|  | SN9816 | 14.97 ± 0.92 | 155.55 ± 5.93 | 5.58 ± 0.51 | 90.57 ± 1.47 | 24.04 ± 0.25 | 13.81 ± 1.04^**^ | 21.42 ± 2.03^**^ |
|  | L-11 | 18.57 ± 1.22^**^ | 204.33 ± 5.49^**^ | 7.75 ± 0.62^**^ | 71.17 ± 0.77^**^ | 26.07 ± 0.29^**^ | 15.84 ± 1.21^**^ | 30.43 ± 2.35^**^ |
|  | L-17 | 17.74 ± 0.94^**^ | 203.11 ± 5.35^**^ | 7.25 ± 0.62^**^ | 71.81 ± 1.30^**^ | 26.02 ± 0.17^**^ | 15.71 ± 1.27^**^ | 31.55 ± 3.18^**^ |
|  | L-26 | 18.28 ± 1.43^**^ | 202.44 ± 9.01^**^ | 7.50 ± 0.52^**^ | 71.36 ± 1.41^**^ | 26.16 ± 0.18^**^ | 14.95 ± 1.69 | 29.83 ± 2.97^**^ |
|  | *osatg8b-1* | 6.35 ± 1.06^**^ | 114.30 ± 9.65^**^ | 2.25 ± 0.45^**^ | 89.62 ± 1.21 | 23.35 ± 0.15 | 8.65 ± 1.03^**^ | 10.82 ± 1.82^**^ |
|  | *osatg8b-2* | 7.38 ± 1.23^**^ | 111.37 ± 7.35^**^ | 3.14 ± 0.78^**^ | 88.93 ± 1.02 | 23.76 ± 0.29 | 9.39 ± 1.61^**^ | 11.74 ± 1.67^**^ |

Values are means ± SD (n = 12), ^**^*P* < 0.01 (*t*-test) indicates the significant differences.
