# Supplementary material for: Autophagy mediates grain yield and nitrogen stress resistance by modulating nitrogen remobilization in rice
Source: PLoS One. 2021 Jan 14;16(1):e0244996. doi: 10.1371/journal.pone.0244996 (PMC7808584; doi:10.1371/journal.pone.0244996)
Supplement: S3 Table — (DOCX) [file pone.0244996.s006.docx]

| **S3 Table. Confirmation of RNA-Seq expression profiles with qRT-PCR.** | | | | | | |
| --- | --- | --- | --- | --- | --- | --- |
| Gene ID | Log_2_ (fold changes) | | | | | |
|  | (L-26 vs SN9816)  NS (225kg·ha^-1^) | |  | | (L-26 vs SN9816)  NL (75kg·ha^-1^) | |
|  | RNA-Seq | RT-qPCR | | RNA-Seq | | RT-qPCR |
| LOC_Os02g03640 | 5.93 | 5.21 | | 5.85 | | 4.72 |
| LOC_Os08g29020 | 5.16 | 4.17 | | 5.28 | | 4.97 |
| LOC_Os04g53240 | 4.23 | 3.71 | | 4.69 | | 3.91 |
| LOC_Os10g25040 | 3.10 | 2.84 | | 3.16 | | 3.41 |
| LOC_Os11g02520 | 2.18 | 1.81 | | 1.63 | | 1.33 |
| LOC_Os11g01010 | 1.24 | 1.53 | | 1.26 | | 1.13 |
| LOC_Os01g57082 | -1.61 | -1.45 | | -2.48 | | -1.91 |
| LOC_Os02g02120 | -1.01 | -0.99 | | -1.85 | | -1.56 |
| LOC_Os02g13430 | -1.07 | -0.68 | | -1.74 | | -1.53 |
| LOC_Os02g32660 | -1.33 | -1.19 | | -1.32 | | -1.44 |
| LOC_Os02g49510 | -2.97 | -2.58 | | -2.91 | | -3.91 |
| LOC_Os02g53180 | -4.27 | -4.83 | | -3.00 | | -4.12 |
| LOC_Os03g62200 | -1.35 | -1.17 | | -1.26 | | -0.87 |
| LOC_Os04g43800 | -1.54 | -1.90 | | -1.41 | | -1.82 |
| LOC_Os05g01140 | -1.12 | -1.47 | | -1.15 | | -1.65 |
| LOC_Os05g11130 | -1.94 | -2.20 | | -1.97 | | -2.65 |
| LOC_Os12g02330 | -1.47 | -1.09 | | -1.66 | | -1.71 |
| LOC_Os12g07210 | -1.68 | -2.00 | | -1.95 | | -1.65 |
