# Supplementary figures and images for: Autophagy mediates grain yield and nitrogen stress resistance by modulating nitrogen remobilization in rice
Source: PLoS One. 2021 Jan 14;16(1):e0244996. doi: 10.1371/journal.pone.0244996 (PMC7808584; doi:10.1371/journal.pone.0244996)

## Uncropped original image files for the immunoblots in Figure 2C

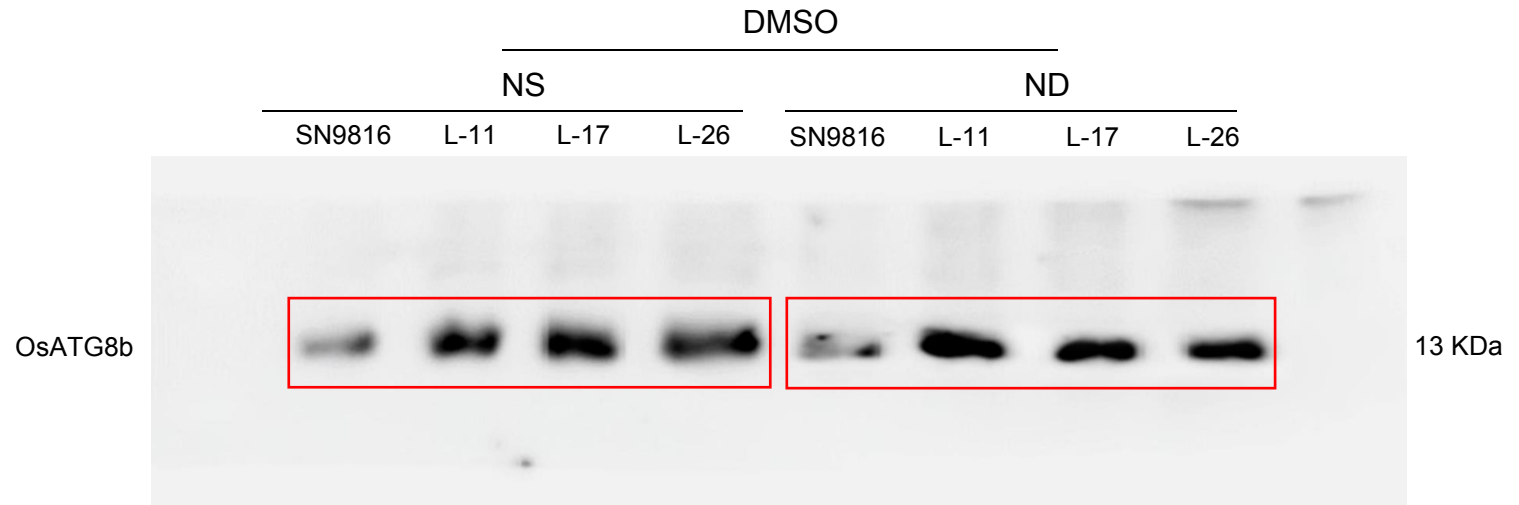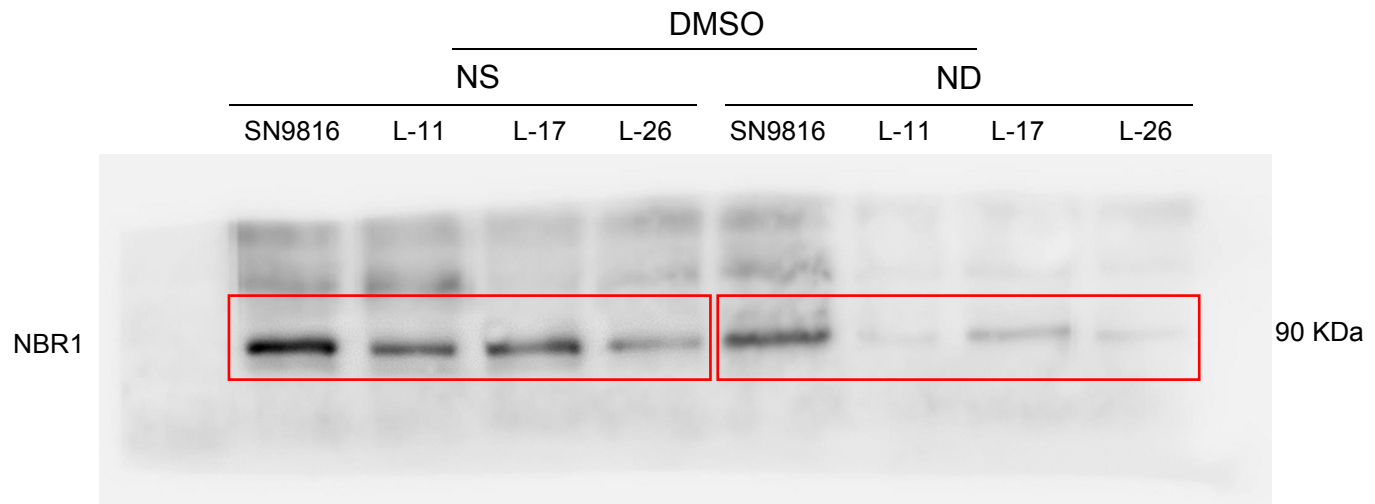

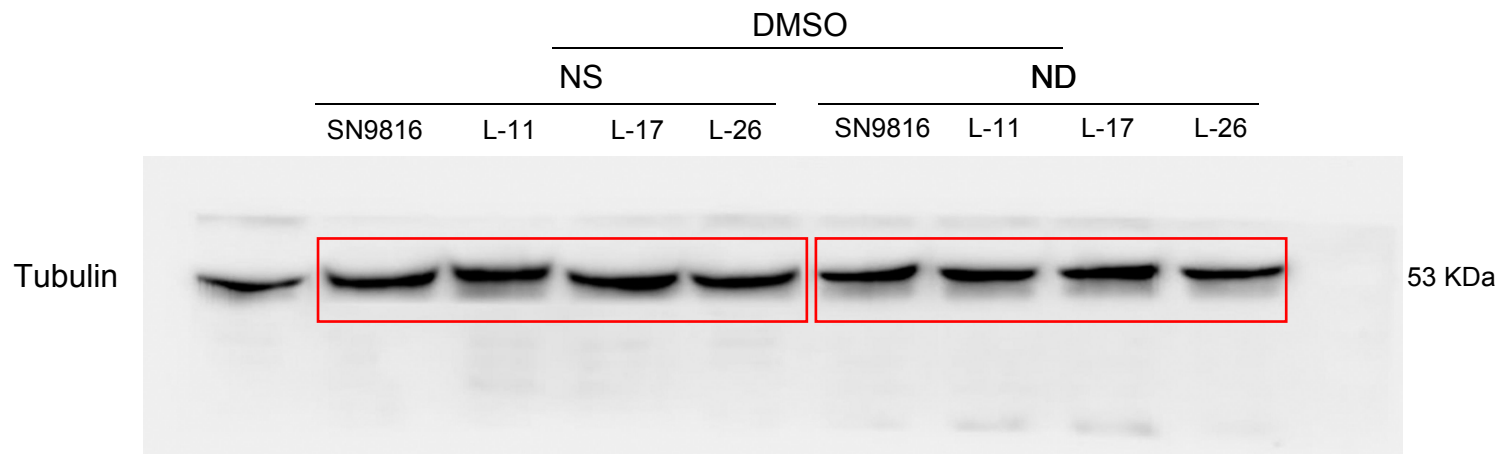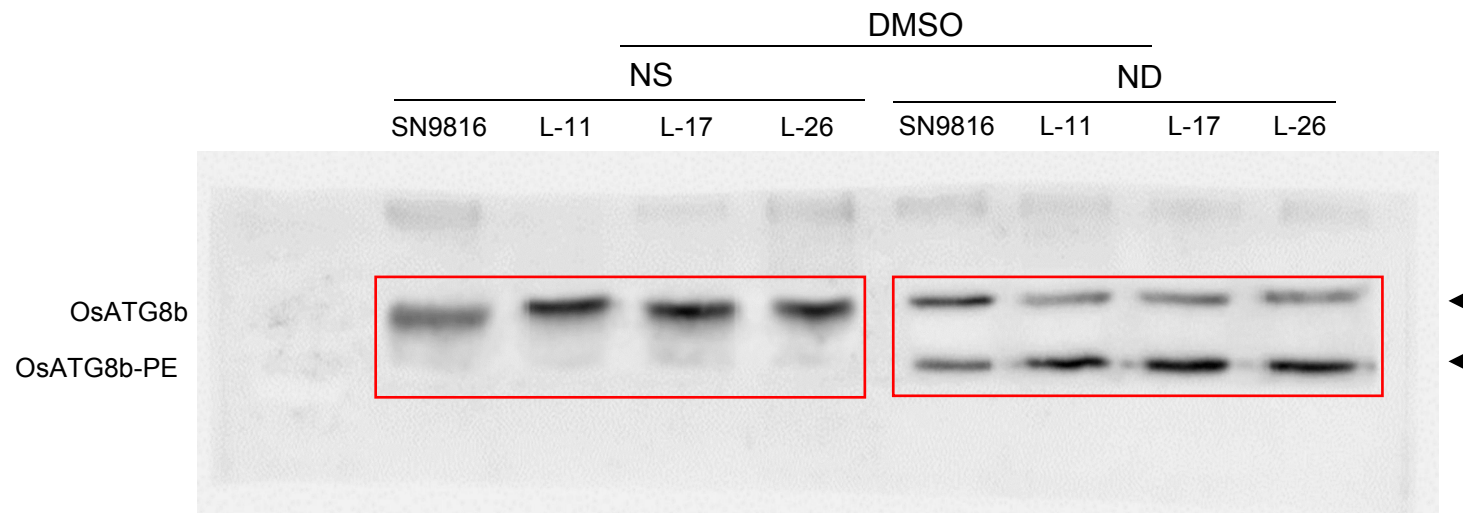

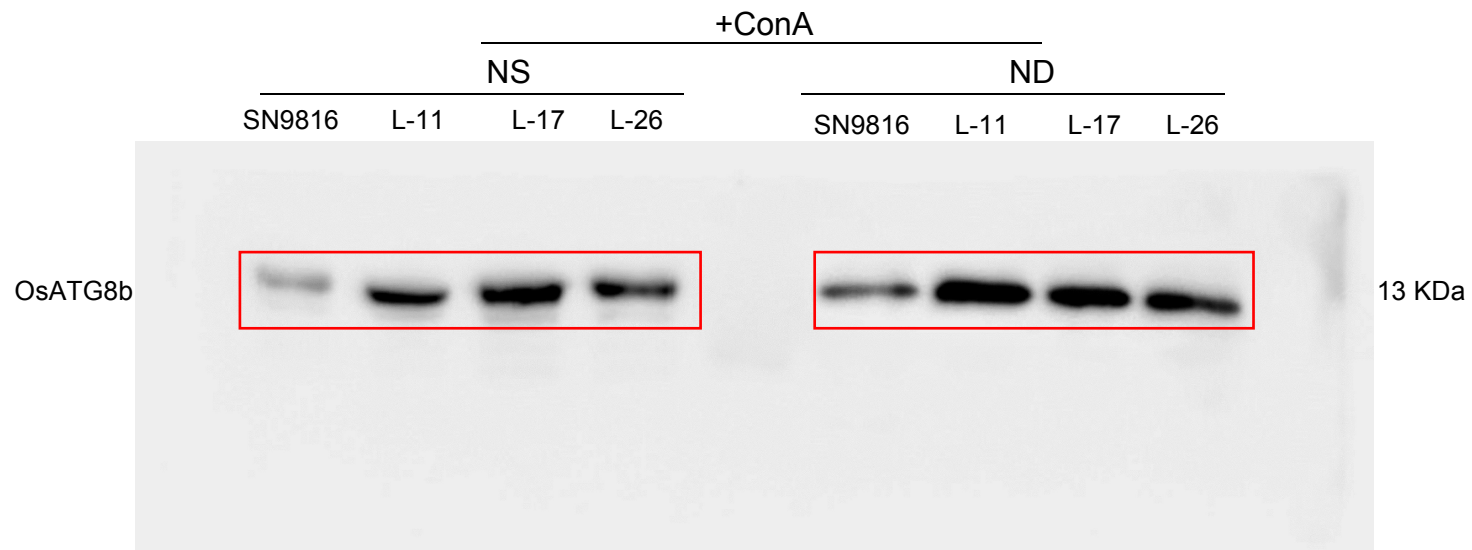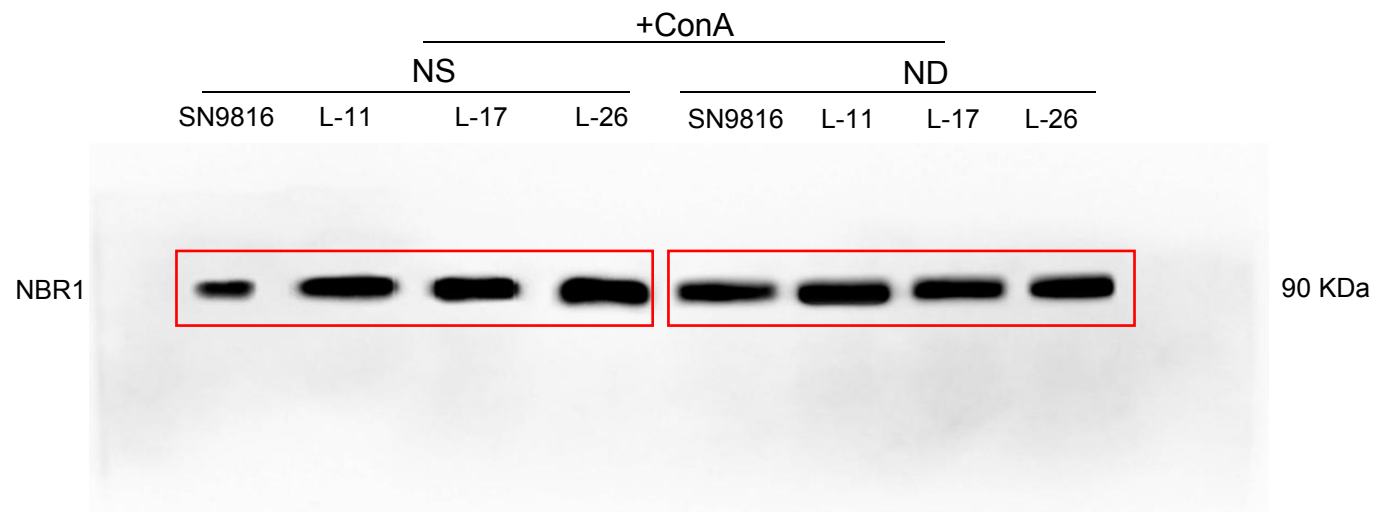

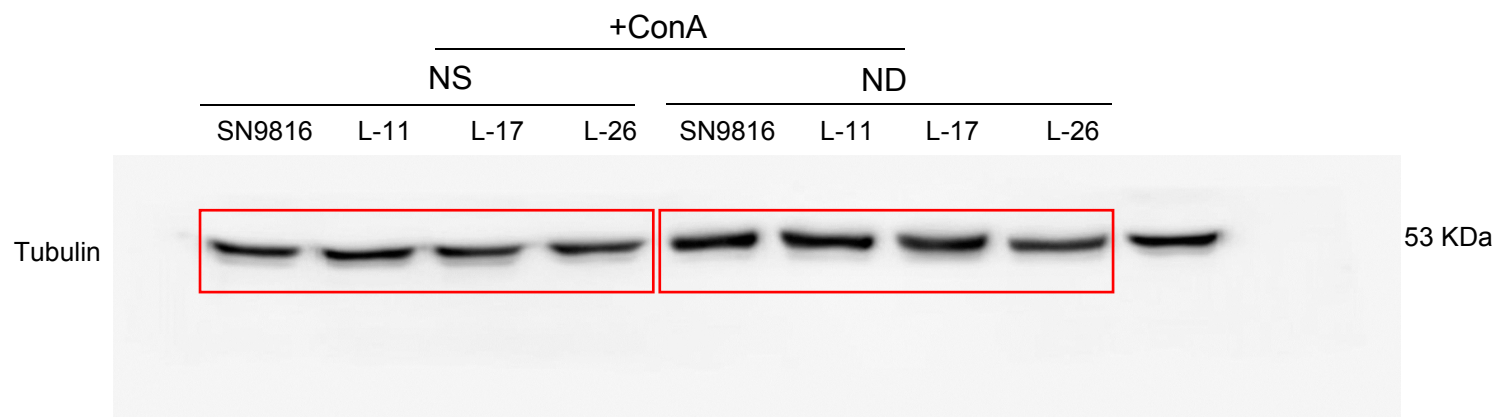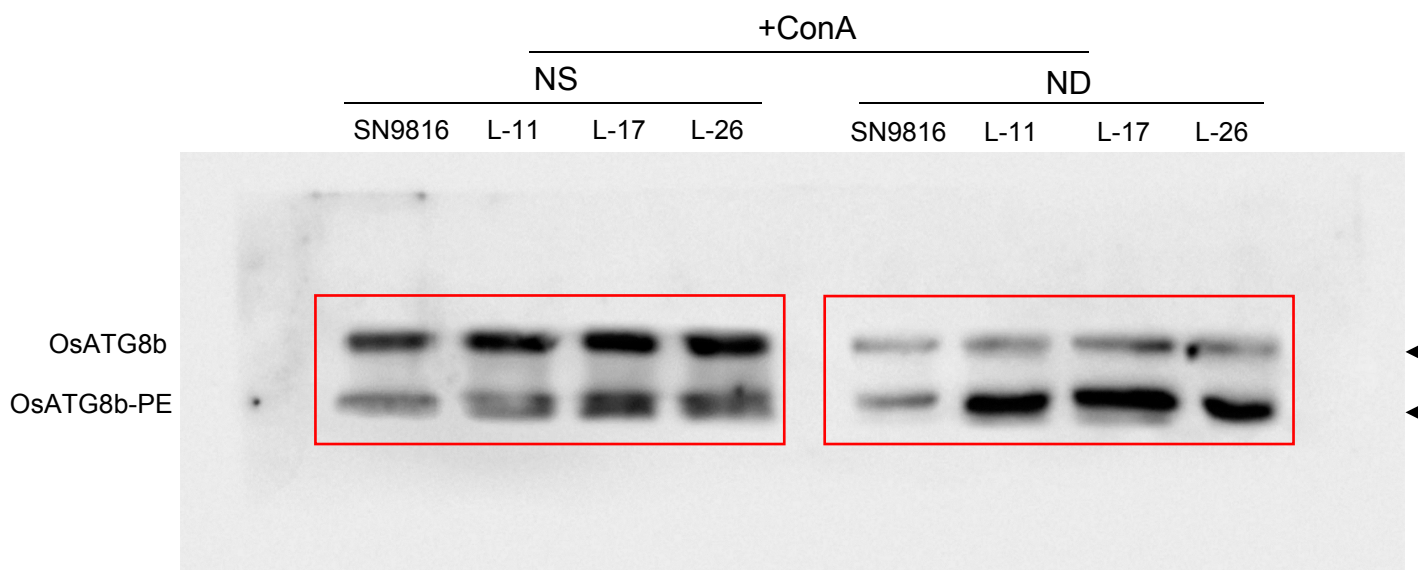

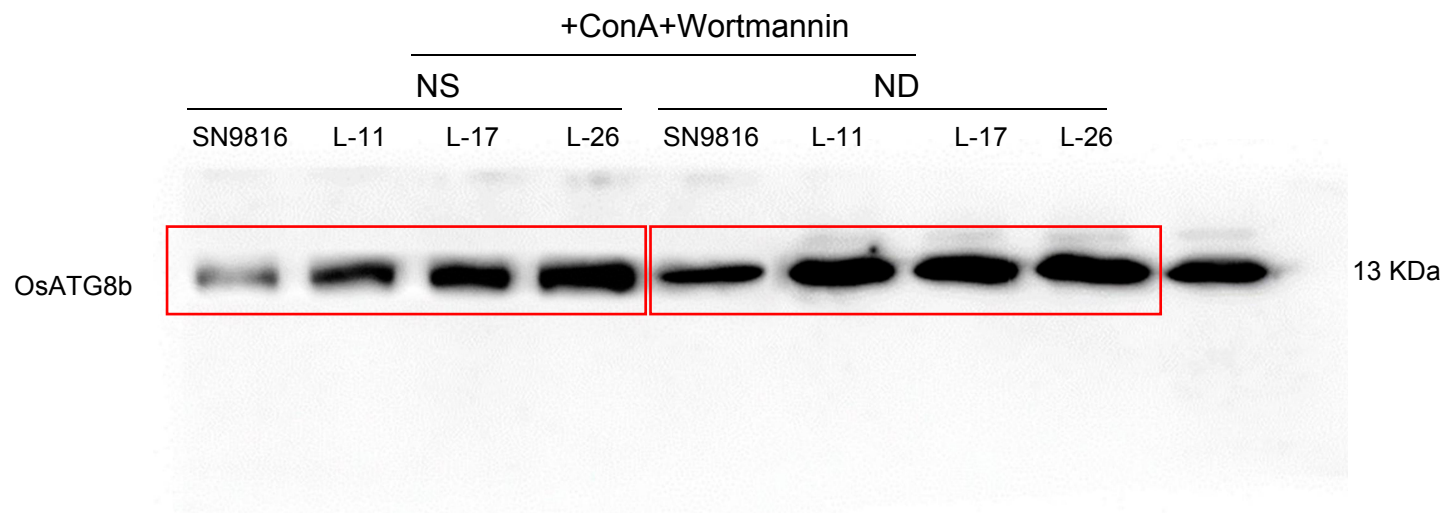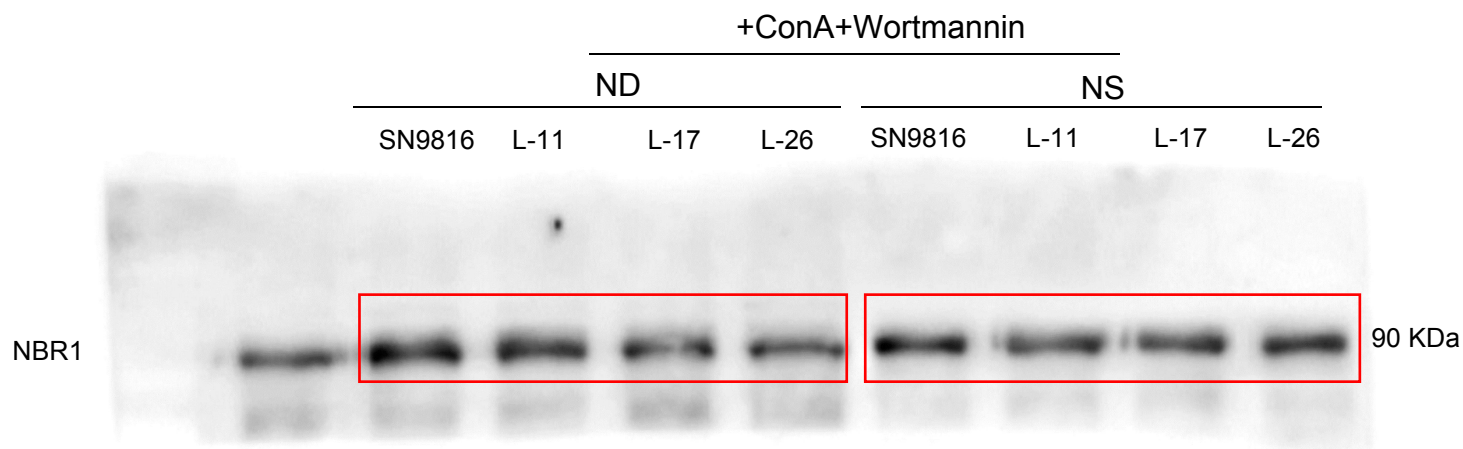

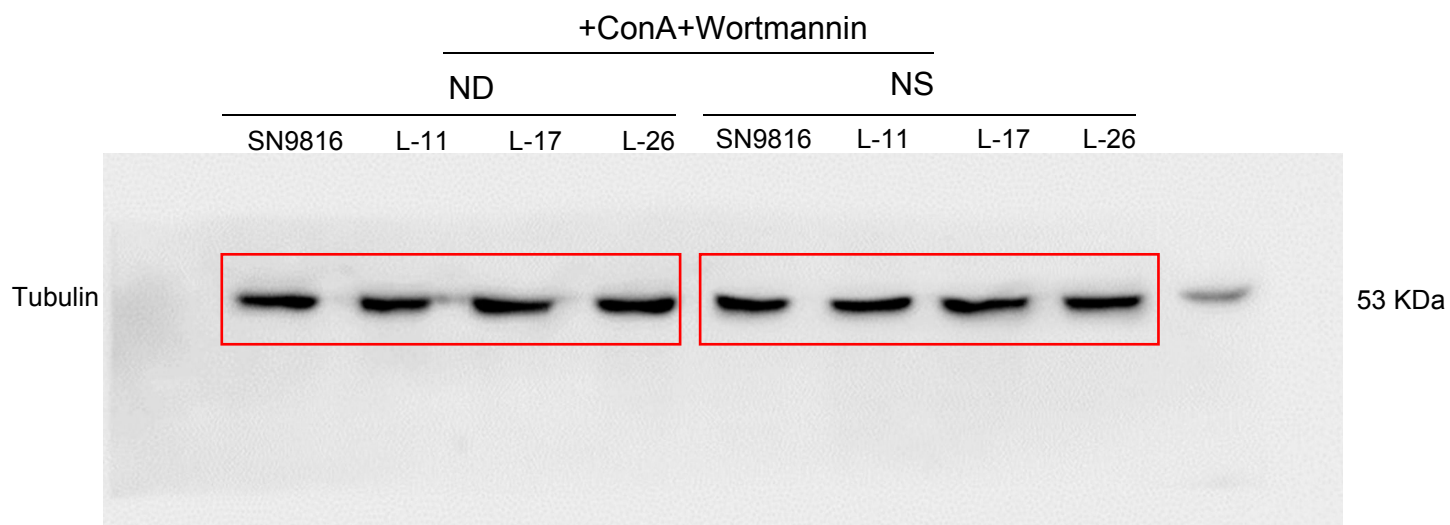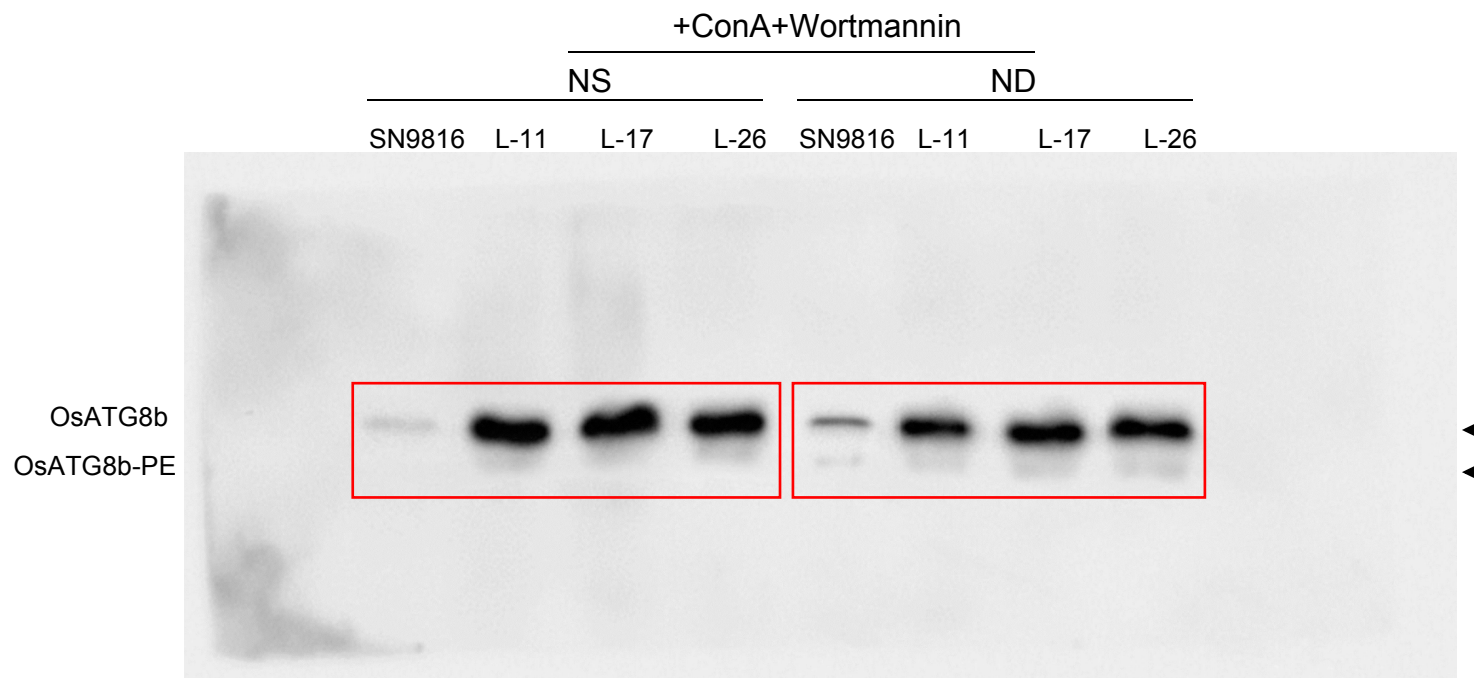

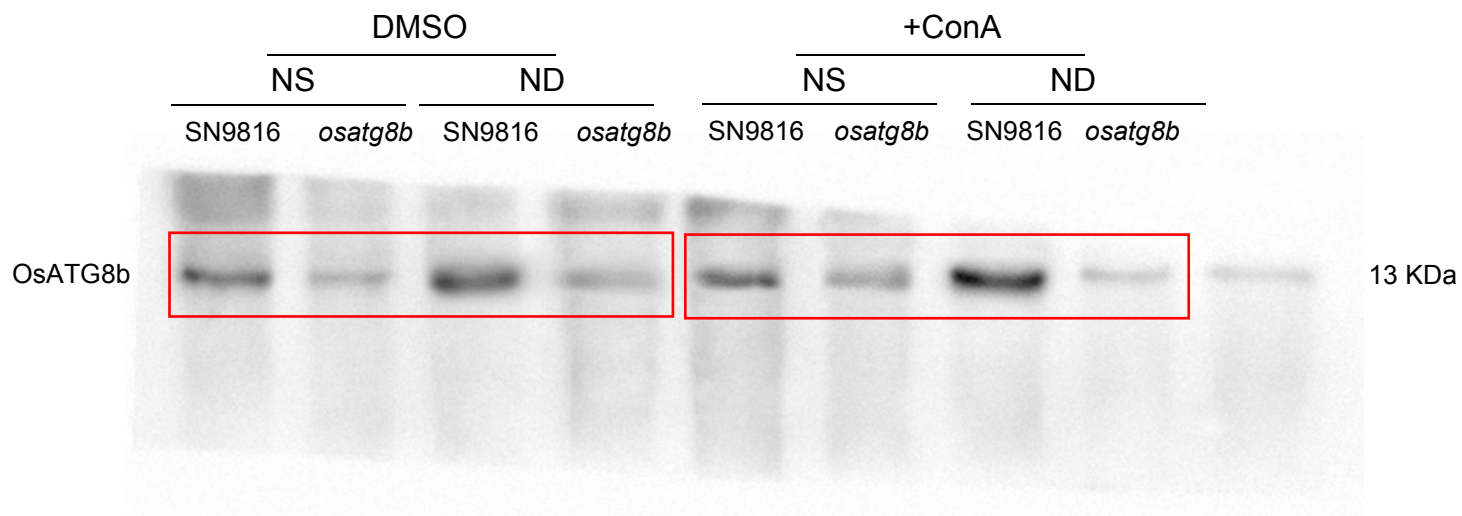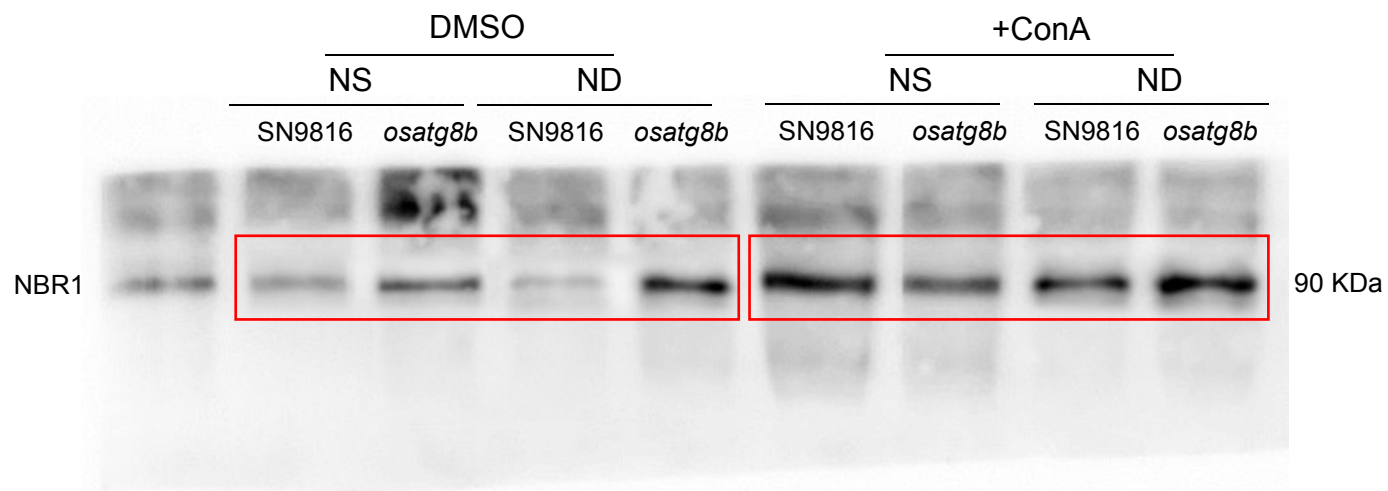

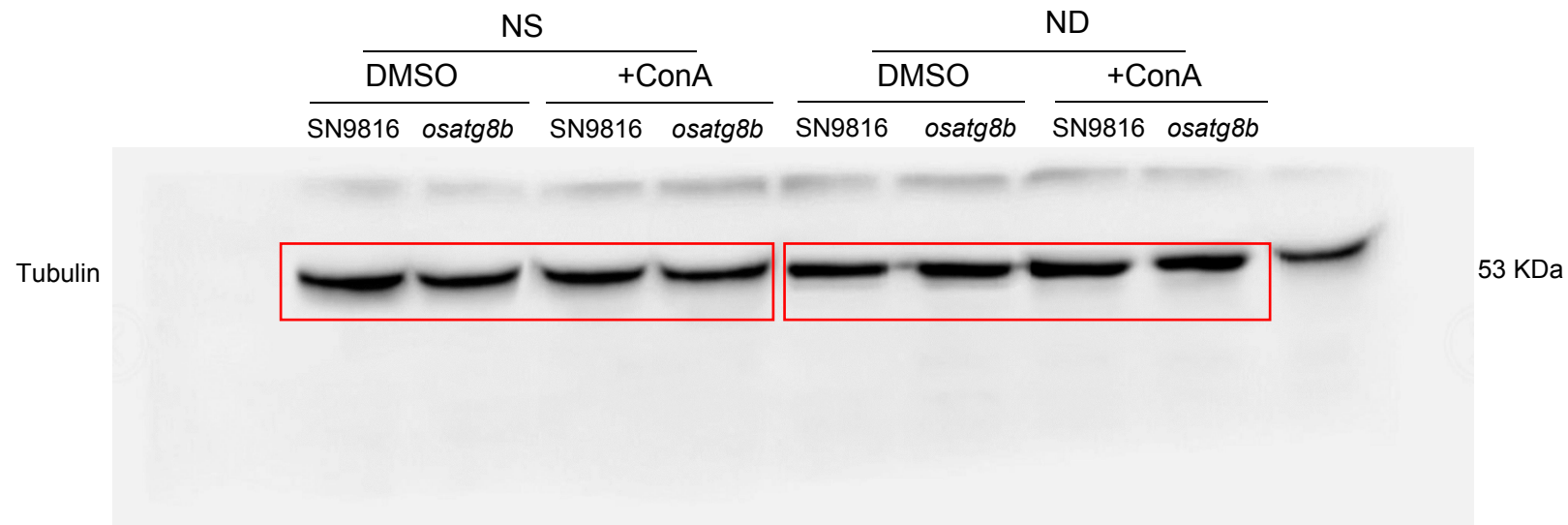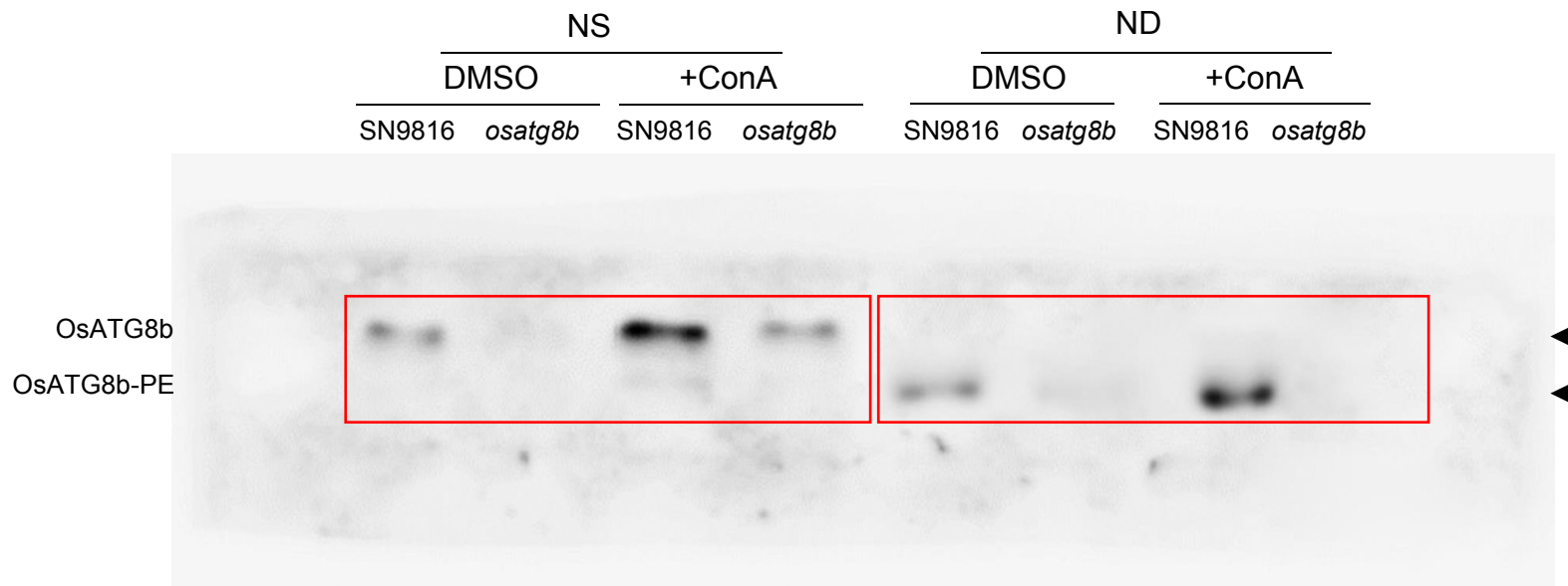

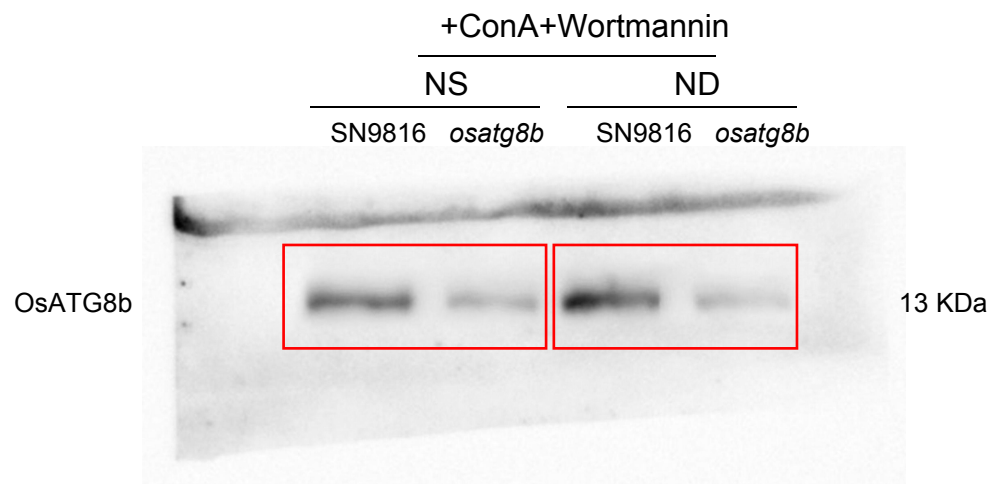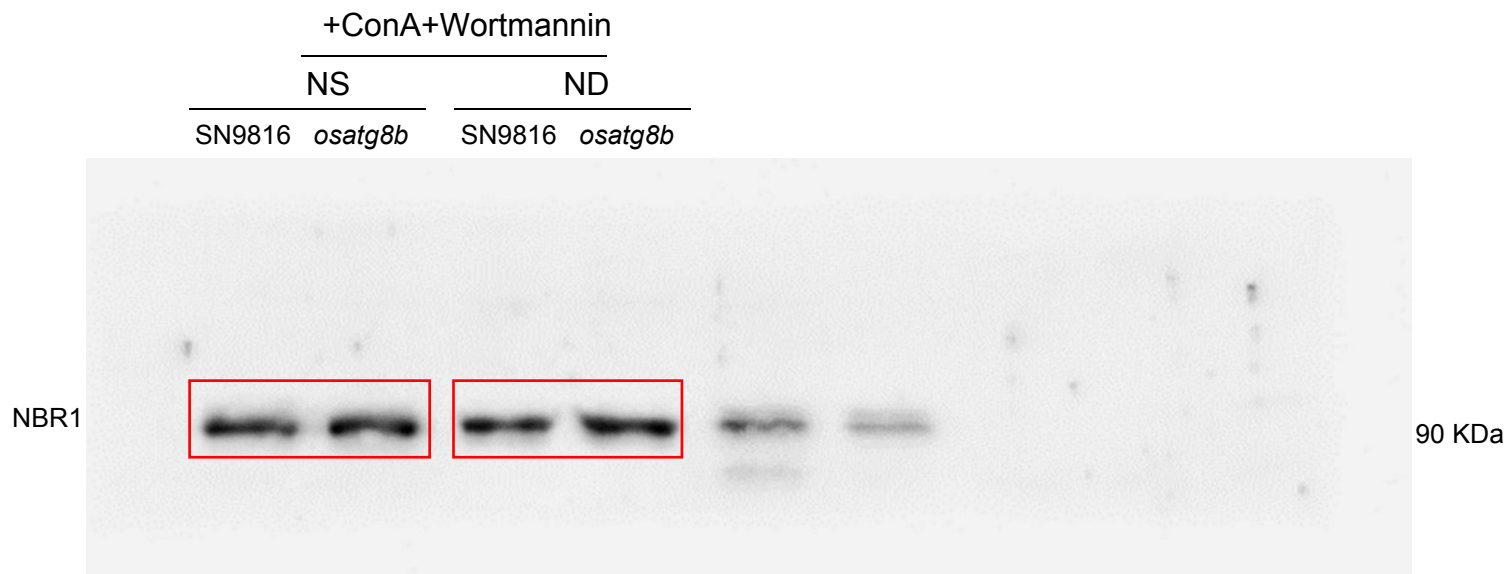

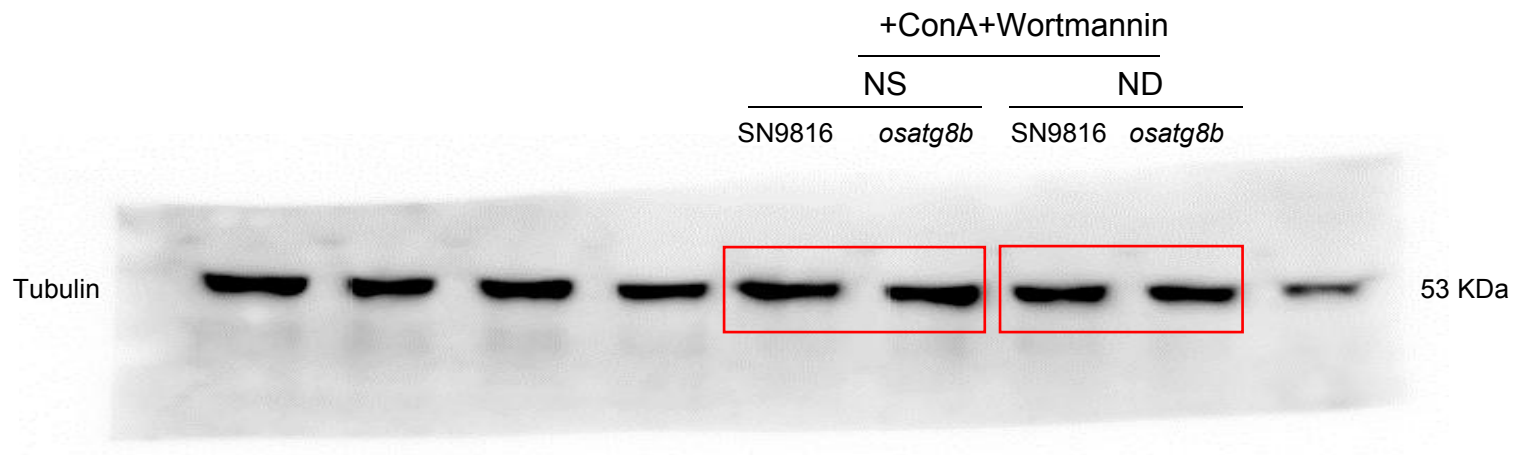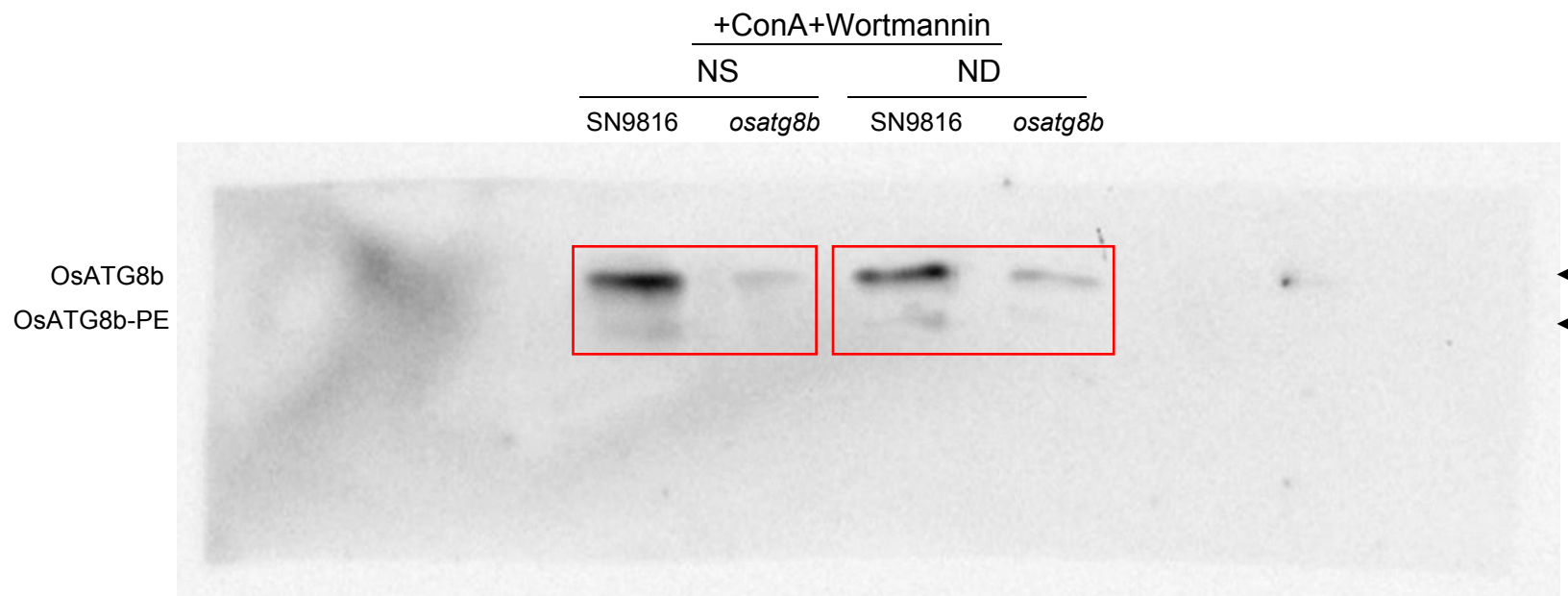

Supplement: S1 Raw images — (PDF) [file pone.0244996.s007.pdf]
